# Lymph nodes are sites of prolonged bacterial persistence during *Mycobacterium tuberculosis* infection in macaques

**DOI:** 10.1371/journal.ppat.1007337

**Published:** 2018-11-01

**Authors:** Sharie Keanne C. Ganchua, Anthony M. Cadena, Pauline Maiello, Hannah P. Gideon, Amy J. Myers, Beth F. Junecko, Edwin C. Klein, Philana Ling Lin, Joshua T. Mattila, JoAnne L. Flynn

**Affiliations:** 1 Department of Microbiology and Molecular Genetics, University of Pittsburgh School of Medicine, Pittsburgh, Pennsylvania, United States of America; 2 Department of Infectious Disease and Microbiology, University of Pittsburgh Graduate School of Public Health, Pittsburgh, Pennsylvania, United States of America; 3 Division of Laboratory Animal Resources, University of Pittsburgh, Pittsburgh, Pennsylvania, United States of America; 4 Department of Pediatrics, Children’s Hospital of Pittsburgh, University of Pittsburgh Medical Center, Pittsburgh, Pennsylvania, United States of America; McGill UniversityHealth Centre, CANADA

## Abstract

Tuberculosis is commonly considered a chronic lung disease, however, extrapulmonary infection can occur in any organ. Even though lymph nodes (LN) are among the most common sites of extrapulmonary *Mycobacterium tuberculosis* (Mtb) infection, and thoracic LNs are frequently infected in humans, bacterial dynamics and the effect of Mtb infection in LN structure and function is relatively unstudied. We surveyed thoracic LNs from Mtb-infected cynomolgus and rhesus macaques analyzing PET CT scans, bacterial burden, LN structure and immune function. FDG avidity correlated with the presence of live bacteria in LNs at necropsy. Lymph nodes have different trajectories (increasing, maintaining, decreasing in PET activity over time) even within the same animal. Rhesus macaques are more susceptible to Mtb infection than cynomolgus macaques and this is in part due to more extensive LN pathology. Here, we show that Mtb grows to the same level in cynomolgus and rhesus macaque LNs, however, cynomolgus macaques control Mtb at later time points post-infection while rhesus macaques do not. Notably, compared to lung granulomas, LNs are generally poor at killing Mtb, even with drug treatment. Granulomas that form in LNs lack B cell-rich tertiary lymphoid structures, disrupt LN structure by pushing out T cells and B cells, introduce large numbers of macrophages that can serve as niches for Mtb, and destroy normal vasculature. Our data support that LNs are not only sites of antigen presentation and immune activation during infection, but also serve as important sites for persistence of significant numbers of Mtb bacilli.

## Author summary

Since tuberculosis is commonly considered a chronic lung disease, most studies in tuberculosis focus on the lungs while lymph nodes are almost always depicted only as sites of antigen presentation and immune activation. However, lymph nodes are among the most frequently infected sites of *Mycobacterium tuberculosis* (Mtb) aside from the lungs. The effect of Mtb infection and how lymph nodes respond to Mtb infection is currently unknown. To investigate this, we examined the lymph nodes of two macaque species, cynomolgus and rhesus macaques, at different time points after Mtb infection. We found that overall lymph nodes are not effective killers of Mtb; the lymph nodes of rhesus macaques being less effective at killing Mtb than cynomolgus macaques. Mtb infection also resulted in the destruction of the lymph node structure and this was associated with increased bacterial burden. After a short course of anti-TB drug therapy, the reduction in bacterial burden was lower in lymph nodes compared to lung granulomas. Our data show that aside from being sites of antigen presentation and immune activation, lymph nodes are also niches of Mtb growth and persistence.

## Introduction

Tuberculosis (TB) is one of the significant causes of morbidity and mortality in the world. It is estimated that 2.3 billion people worldwide are infected with *Mycobacterium tuberculosis* (Mtb), the causative agent of TB. In 2016, there were an estimated 10.4 million new TB cases and 1.6 million deaths [1]. Although the most common site of infection and disease is the lungs, extrapulmonary TB also occurs, and lymph nodes (LN) are the most common sites of extrapulmonary Mtb infection [2, 3]. In humans, it has been classically observed that a tuberculous pulmonary lesion is almost always accompanied by a granulomatous thoracic LN; this is called a Ghon complex [4, 5]. Moreover, reports of TB-associated cervical lymphadenitis (scrofula) span from antiquity to the present [6–8]. Historically, it was not known whether scrofula was related to TB [6], but some individuals with scrofula eventually died from TB-associated disease including TB meningitis and pneumonia, suggesting a relationship between these dissimilar-appearing pathologies [6–8] and this has been demonstrated by more modern techniques, including microscopy, culture and nucleic acid amplification tests [9, 10].

Lymph node infection occurs in people with active and clinically latent TB [11–14] but the timing and frequency of LN infection, bacterial load in LNs, and the relationship between LN infection and disease outcome remain uncertain. Most studies on Mtb infection in LNs were in murine models and focused mainly on identifying the mechanisms and regulation of T cell priming in LNs [15–18]. However, mice are different from humans in that they only have a single lung-draining mediastinal LN. Moreover, pathologic presentation and course of TB in mice is substantially different than that seen in humans [19], and there has been little emphasis on identifying the long-term consequences of Mtb infection in these lymphoid tissues. Guinea pigs have been shown to develop rapid and severe lymphadenopathy after Mtb infection with live bacteria and T cell influx demonstrable in LNs as early as 5 days post-infection [20–22]. In cattle, LNs are the most common site of *M*. *bovis* infection with microscopic lesions visible as early as 7 days post-infection [23–25]. In a small study examining the prevalence of lung and LN infections in bovine TB, all cattle had *M*. *bovis*-infected LNs while pulmonary infection was much less common (1 of 15 cattle) [26]. However, since tuberculous lung lesions can be small, some authors as cited by Neill et al, believe that without thorough examination these lesions are missed [25]. It is widely believed that LNs get infected with *M*. *bovis* first while lung lesions develop later during infection [24, 25].

Rhesus and cynomolgus macaques have been used as models of human TB, and represent the spectrum of pathology and disease outcome seen in human Mtb infection [27–30]. Thoracic LNs are frequently infected in Mtb-infected macaques [27, 28, 31] and the first signs of reactivated TB assessed by microscopic histology can occur in thoracic LNs. We previously showed that macaques considered high risk for reactivation after TNF neutralization had a greater proportion of thoracic LN with Mtb growth compared to those that were low risk [32]. Moreover, in studies where immune suppression was induced by anti-CD4 antibodies, reactivation was associated with macaques with greater depletion of CD4 T cells in thoracic LNs [32, 33], suggesting immune responses in these organs are important for overall protection. Anti-TNF induced reactivation can also present in thoracic LNs [32]. In BCG or BCG+H56 vaccinated cynomolgus macaques, protection against reactivation was associated with limited LN involvement [34]. Although closely related to cynomolgus macaques, rhesus macaques are more susceptible to TB owing in part to their more extensive LN pathology [35]. Rhesus macaques have increased numbers of Mtb-infected LNs, higher bacterial burden per LN, and greater LN pathology than cynomolgus macaques. Extensive LN disease can lead to enlargement of LNs such that they impinge on the macaque’s airway, occasionally leading to lung lobe collapse. Enlarged and necrotic LNs have also been noted to erode into the airways leading to substantial dissemination [35].

Although it is clear that LNs are commonly infected, we know very little about how Mtb infection influences LN structure and function. Lymph nodes are highly-structured organs where T cells and B cells interact with dendritic cells (DCs) in spatially-distinct anatomic regions, and this delicately balanced organization facilitates priming and adaptive immunity [36–39]. Other elements in LNs that are required for proper function and are susceptible to disruption by Mtb infection include subcapsular macrophages and fibroblastic reticular cells [40, 41], conduit systems that mediate fluid flow and antigen entry into LNs, capillaries, and lymphatic vessels [42–44]. Lymphatic endothelial cells have also been shown to promote or restrict Mtb replication depending on their activation status, thus these cells may represent an underappreciated intra-lymph node niche for Mtb [45, 46]. In addition to priming adaptive immunity, LNs have intrinsic antimicrobial capacities that limit dissemination of pathogens [47–49] although it is unknown whether they have this capacity in Mtb infection.

Studies on LNs in TB often focus on diagnosis [9, 50–53] or priming of adaptive immunity [16–18, 36, 54], and despite more than a century of TB research, there are many aspects of the infection that remain unclear. Basic questions including the dynamics of LN Mtb infection, whether LNs are sites of successful defense against Mtb growth, the proportion of LNs that get infected, and how Mtb infection affects LN structure and function remain unanswered. To address these questions, we performed a comprehensive study of thoracic LNs in Mtb-infected cynomolgus and rhesus macaques to identify how Mtb infection changes LNs. We found that LNs in Mtb-infected macaques increased in PET-CT-measured metabolic activity early during infection, and at necropsy, almost all of the FDG-avid LNs contained viable Mtb or persistent Mtb DNA. We show that Mtb grows to the same level in LNs of both macaque species, however, cynomolgus macaque LNs were better able to control the infection compared to rhesus macaque LNs. In comparison with granulomas in the lung [55], thoracic LNs had limited abilities to kill Mtb. Granulomas that form in Mtb-infected LNs disrupt LN structures and greater destruction of the LN structure is associated with higher Mtb burden. Our data support that LNs are a niche for persistent infection, and are likely to play a larger role in the pathogenesis of TB than previously appreciated. Moreover, identifying relationships between LNs, bacterial persistence, and disease progression may yield new insights into disease pathogenesis, improve TB treatment and limit reactivation of latent TB.

## Results

### ^18^F-FDG PET CT imaging can identify Mtb-infected thoracic lymph nodes

**Fig 1:**
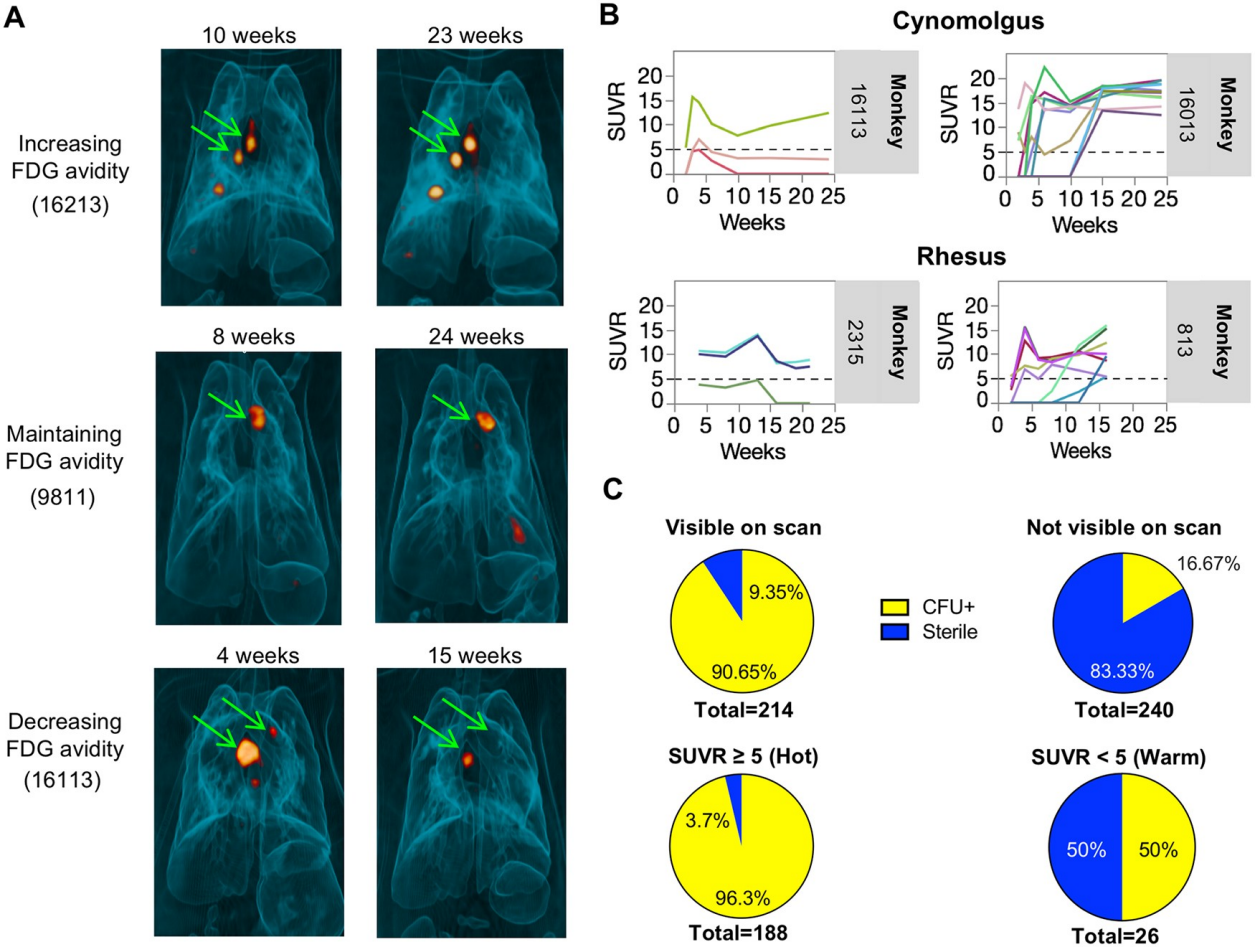
FDG PET CT analysis of Mtb infection in lymph nodes of cynomolgus and rhesus macaques. A. PET/CT scans of 3 different macaques (monkey numbers 16213, 9811, 16113 showing different trajectories of thoracic lymph nodes at different time points post infection. B. Representative serial PET CT FDG SUVR plots showing several lymph nodes visible by PET at 2 weeks post infection in four different animals. Trajectories of individual lymph nodes in an animal is shown to be independent of each other. Each line is a lymph node. Dotted line represents the cut-off for calling FDG+ LNs “hot” (SUVR≥5). C. Most lymph nodes visible (SUVR≥2.3) on scan by PET 1–2 days before necropsy harbor live Mtb (top left panel), while only a small proportion of those that are not seen by PET have live Mtb (top right panel). Most”hot” lymph nodes (SUVR≥5) were CFU+ compared to only half of “warm” lymph nodes (SUVR 2.3–4.99) (bottom panels).

Macaques have multiple thoracic LNs [28] that drain different lung regions, and variable numbers among animals. We previously described differences in the extent of LN disease between rhesus and cynomolgus macaques [35]. To address the overall question of LN infection dynamics and differences between species, we first used PET CT imaging to track inflammation in thoracic LNs following Mtb infection. Our PET probe was ^18^F-fluorodeoxyglucose (FDG), a radiolabeled glucose analog that is taken up by metabolically active cells; we have demonstrated previously that FDG avidity is enhanced in Mtb-affected tissues and is a surrogate for inflammation in granulomas and lungs [34, 35, 56–59]. In our study, LNs that were FDG avid and detectable by PET follow one of three courses of FDG uptake over the course of the infection: increased, maintained, or decreased FDG avidity (Fig 1A and 1B). In many macaques, one or more thoracic LNs had measurable FDG uptake by 2 weeks post-infection (examples shown in Fig 1B), and different thoracic LNs within an animal followed different trajectories (ie. increasing, decreasing, maintaining FDG uptake) such that one animal could have individual LNs with different uptake patterns (Fig 1B). To examine the relationship between FDG positivity and the presence of viable Mtb in a specific LN, we performed PET CT scans on animals 1–2 days prior to necropsy and cultured LN homogenates to measure bacterial burden. We found 90.65% (194 of 214) of LNs visible on scan by PET at necropsy contained culturable Mtb at necropsy, while 83.33% (200 of 240) of undetectable LNs (SUVR = 0) were sterile. Of the “hot” lymph nodes (defined as SUVR ≥ 5 [34]) on the pre-necropsy PET CT scan, 96.3% (181 of 188) of thoracic LNs contained culturable Mtb, while in thoracic LNs that were “warm” (detectable but with SUVR< 5), only 50% (13 of 26) contained culturable Mtb (Fig 1C). There was a modest correlation (Spearman’s rho = 0.4812, p < 0.001) between SUVR and live bacterial burden in PET-detectable LNs at necropsy (S1 Fig). These data suggest that mycobacterial involvement in thoracic LNs is a dynamic process and PET CT-detected FDG uptake is associated with bacterial infection in LNs.

### Thoracic lymph nodes of rhesus macaques have reduced killing capacity for Mtb compared to cynomolgus macaques

**Fig 2:**
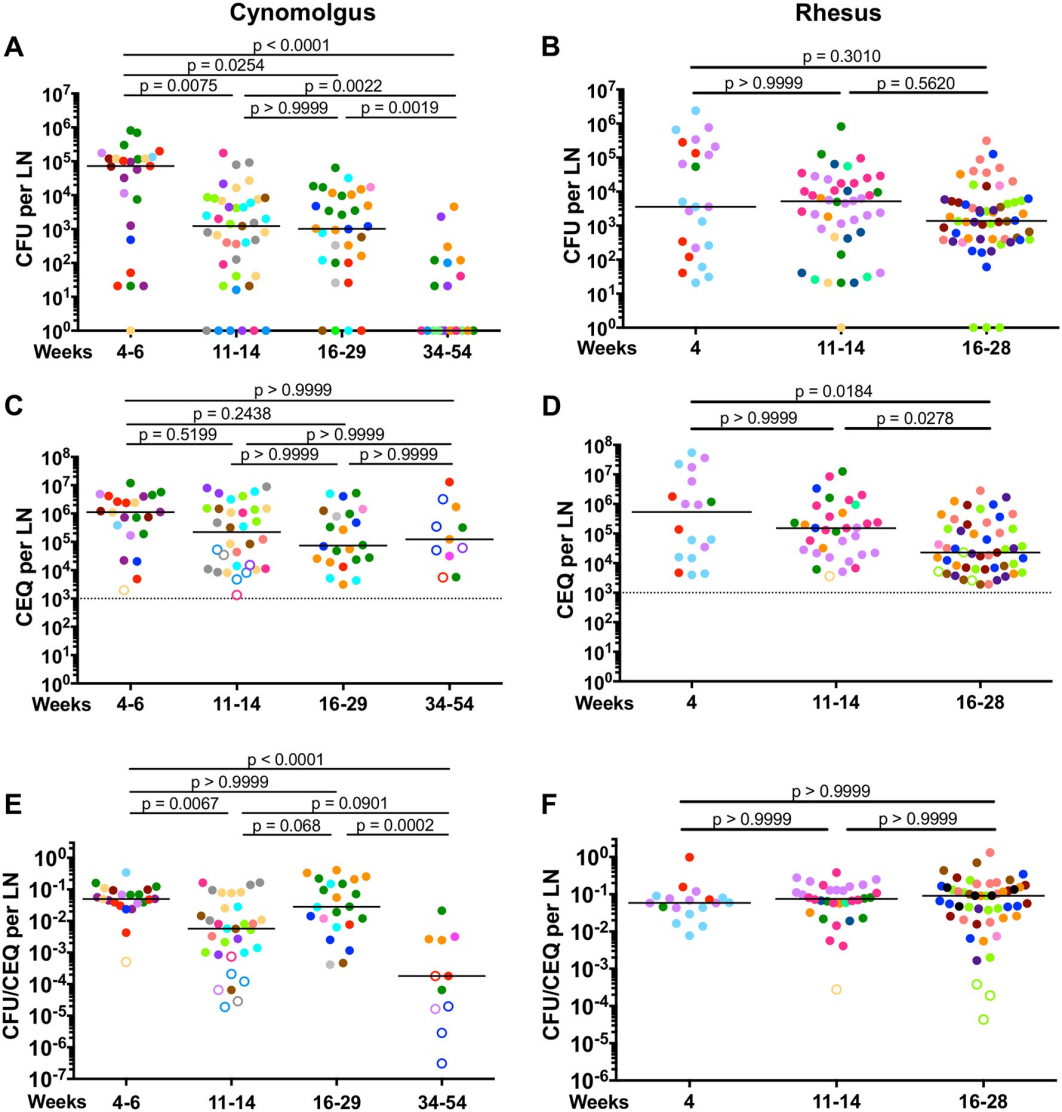
Mtb burden and killing in lymph nodes of cynomolgus and rhesus macaques. A, B. Live Mtb burden (CFU) in thoracic LNs from cynomolgus (A) and rhesus (B) macaques at various time points post-infection (at necropsy). Lymph node CFU of cynomolgus macaques decreases over the course of infection while rhesus macaques do not. C, D. Total (live+dead, CEQ) Mtb burden in cynomolgus (C) and rhesus (D) macaque LNs. There was no difference in the level of CEQ in cynomolgus macaque LNs over the course of infection, while a decline in CEQ was found in rhesus macaque LNs at later time points post-infection. E, F. Mtb killing in thoracic lymph nodes, as calculated as the ratio of live (CFU) compared to total (CEQ) bacteria. Cynomolgus macaque LNs (E) exhibit poor Mtb killing at 4 weeks post infection but improve over the course of infection. Highest Mtb killing capacity was observed in monkeys with latent infection (34–54 weeks post infection). Little killing was observed in rhesus macaque LNs (F). The CFU was transformed by adding 1 to reflect sterile LNs with CEQ and/or granulomas either by gross or microscopic examination. For C-F, only LNs in which CEQ were detected were included. Each macaque is shown in a different color. Each data point is one lymph node. Open symbols are sterile lymph nodes (CFU-). The number of macaques per time point post-infection is as follows: a.) 4–6 weeks (Cynos n = 8, Rhesus n = 4); b.) 11–14 weeks (Cynos n = 9, Rhesus n = 7); c.) 16–29 weeks (Cynos n = 9, Rhesus n = 8); d.) 34–54 weeks (Cynos n = 6). The number of lymph nodes analyzed ranged from 4 to 13 per macaque. Dotted line represent the limit of detection of our qPCR assay. Statistics are Kruskal-Wallis with post-hoc Dunn’s multiple test comparisons; p values are shown on figure.

We previously determined that there is variation in the number of thoracic LNs that become infected with Mtb among macaques and differences in LN pathology and total LN bacterial burden between cynomolgus and rhesus macaques [35], however, we know little about bacterial dynamics in individual LNs. To address this, we first determined the number of viable Mtb per LN in both macaque species over time. In cynomolgus macaques, CFU peaked at 4–6 weeks (median = 72001) post-infection which then greatly decreased by 11–14 weeks (median = 1226) and 16–29 weeks (median = 1021) post-infection (Fig 2A). Cynomolgus macaques with well-controlled (clinically latent, [28]) infection necropsied at 34–54 weeks post infection had the lowest median CFU (1 CFU per LN) (Fig 2A). Unlike cynomolgus macaques where CFU per LN decreased over the course of infection, rhesus macaques had relatively stable median CFU per LN (Fig 2B). At both early (4–6 weeks) and later (16–28 weeks) time points post-infection, there was no significant difference between the two species (S2A Fig). Of note, the 16–29 week post-infection group consisted of two animals with active disease (n = 2; 15 LNs; median = 4901 CFU; green and orange circles) and six macaques that were controlling infection (n = 6; 14 LNs; median = 101 CFU) (S3A Fig). A subanalysis showed that there was no significant difference between the LN CFU of the two cynomolgus macaques with active disease and the rhesus macaques at 16–29 weeks post-infection. However, median LN CFU of the six cynomolgus macaques controlling the infection was significantly lower compared to rhesus macaques at this time point (S3A Fig). Moreover, CFU of cynomolgus macaques at 11–14 weeks post infection was significantly lower than rhesus macaques, although the actual difference between medians was only 4-fold (S2A Fig). These data suggest that after reaching a peak in CFU, cynomolgus macaques are capable of reducing the bacterial burden in their LNs over the course of infection while rhesus macaques are not.

To determine whether there were differences in the total (both live and dead) bacterial burden in these LNs, we used a qPCR-based technique amplifying *sigF*, a single-copy mycobacterial gene, to estimate the mycobacterial chromosomes per LN (expressed as chromosomal equivalents [CEQ]) [55]. Chromosomal DNA (CEQ) was first shown to persist in mice lungs after killing Mtb with isoniazid treatment [60]. We previously assessed CEQ in macaque lung granulomas and demonstrated that CEQ persist after bacteria are killed by host responses or by isoniazid [55, 60]. To confirm this technique worked in LNs, we performed qPCR and bacterial culture on LN samples from isoniazid-treated macaques (n = 4) and found similar CEQ numbers in the drug treated and control groups (S4A Fig). Moreover, similar levels of CEQ were found between CFU+ LNs and sterile LNs with granulomas from drug-treated macaques (S4B Fig). This shows that Mtb DNA persists in LNs even after Mtb is killed by isoniazid treatment. As a negative control and to confirm the specificity of our probes, we used LNs from an uninfected macaque and were unable to detect any Mtb genomes in these samples.

We did not find significant differences in CEQ across different time points post-infection in cynomolgus macaques (Fig 2C). However, we saw a reduction in CEQ levels in rhesus macaques at later time points post-infection (4 weeks vs 16–28 weeks, 23.5-fold, p = 0.0184; 11–14 weeks vs 16–28 weeks, 6.7-fold, p = 0.0278) (Fig 2D), which could be because the macaques that have severe disease and have deteriorated clinically are necropsied prior to this time point, and thus the samples are from those with less severe disease. Both species had similar levels of CEQ at 4–6 weeks and 11–14 weeks post-infection. Rhesus macaques at 16–28 weeks post-infection had lower CEQ compared to cynomolgus macaques (3.2-fold) (S2B Fig). These data suggest that Mtb replicates and grows to the same extent in LNs of both species and the lower viable Mtb burden in cynomolgus macaque LNs was not due to fewer Mtb in these tissues, but more likely to those LNs being better able to kill Mtb.

To estimate the ability of LNs to kill Mtb, we evaluated the ratio of live Mtb burden (CFU) and total (live+dead) Mtb burden (CEQ) per LN [55], as we previously described in macaque lung granulomas [55]. As validation of this technique in LNs, we estimated the CFU/CEQ killing ratio in sterile LNs (with evidence of previous infection, i.e. granuloma) compared to CFU+ LNs with granulomas following isonaized treatment (S4C Fig). As expected, isoniazid treatment reduced the CFU/CEQ ratio (i.e. increased bacterial killing) in LNs. Thus, this technique can estimate Mtb killing in lymph nodes in the setting of drug treatment.

Cynomolgus macaque LNs showed little to no killing at 4 weeks post infection, but their ability to kill Mtb increased ~9-fold by 11–14 weeks post infection (Fig 2E). There was no significant difference in the killing capacity of LNs between 4 weeks and 16–29 weeks post-infection. Lymph nodes in the 16–29 weeks group represented macaques with a wide range of disease and the poor killing capacity is largely driven by samples from two monkeys with severe disease at this time point (n = 2; 12 LNs; median = 0.122; green and orange circles vs controlling animals n = 6; 10 LNs; median = 0.0076). We found no significant difference between the killing capacity of cynomolgus macaque LNs who had active disease and rhesus macaque LNs at this time point. However, LNs from cynomolgus macaques who were controlling the infection had higher bacterial killing compared to rhesus macaques (S3B Fig). Lymph nodes from cynomolgus macaques with well-controlled infection sampled at 34–54 weeks had the highest level of bacterial killing (277-fold increase in killing capacity relative to macaques sampled at 4–6 weeks post infection) (Fig 2E). While the Mtb killing capacity of LNs from cynomolgus macaques who are controlling the infection improves over time, rhesus macaque LNs demonstrated little to no Mtb killing at any time point examined (Fig 2F). Cynomolgus macaque thoracic LNs were 13-fold (p < 0.0001) better at killing Mtb than rhesus macaque LNs at 11–14 weeks post-infection (S2C Fig), suggesting that rhesus macaque LN’s reduced ability to kill Mtb may contribute to the more severe LN disease in rhesus macaques during Mtb infection [35].

**Fig 3:**
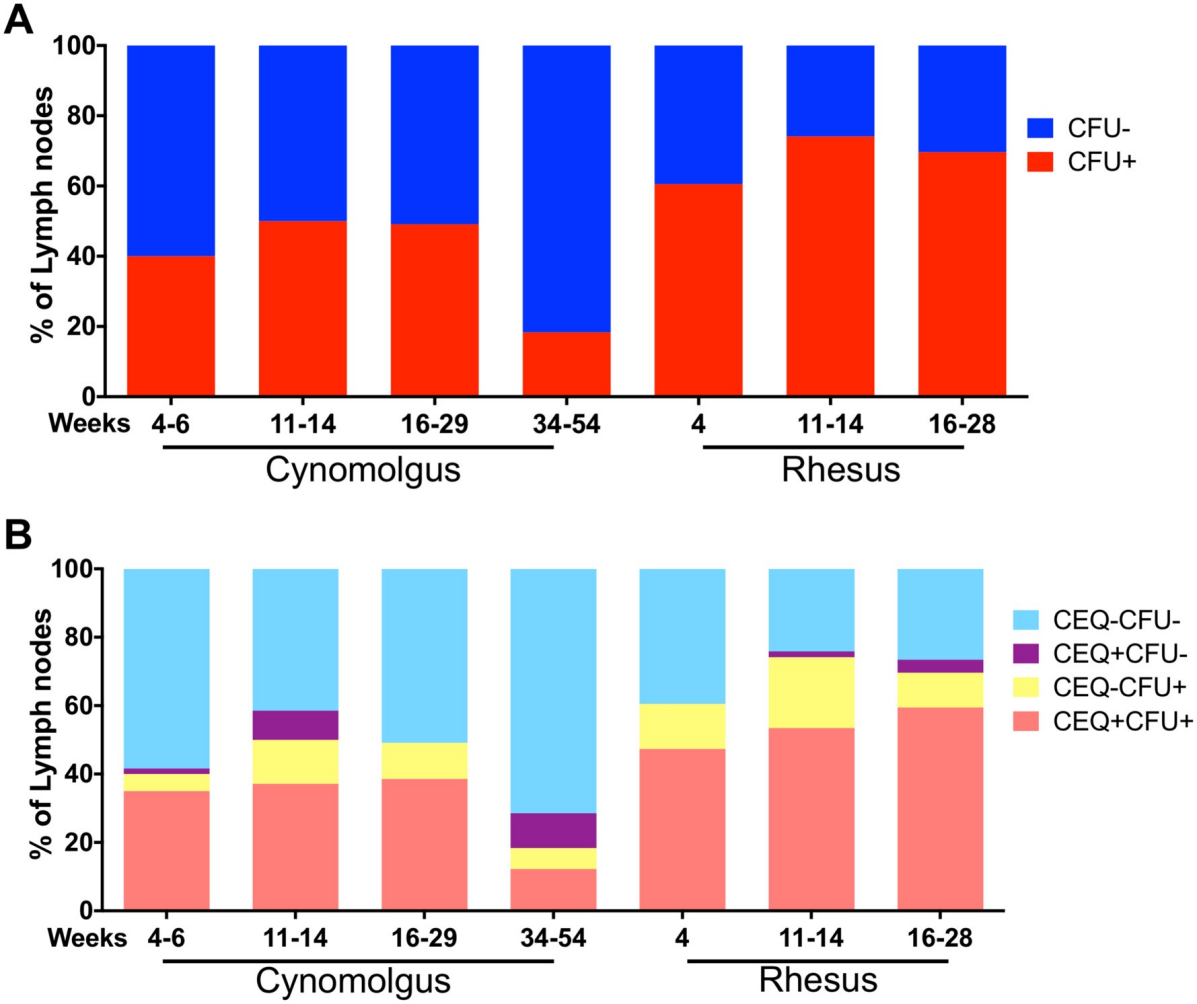
Proportion of thoracic lymph nodes infected with Mtb in cynomolgus and rhesus macaques at necropsy. A. Percent of thoracic LNs that were CFU+ (red) or CFU- (blue) at necropsy. Rhesus macaques had higher proportion of Mtb-infected lymph nodes than cynomolgus macaques. B. Proportion of lymph nodes that were uninfected (CFU-/CEQ-, light blue), infected but were sterile (CEQ+/CFU-, purple), had culturable Mtb but no detected genome (CFU+/CEQ-, yellow) and with culturable Mtb and Mtb genome (CFU+/CEQ+, pink). Threshold for detection of CEQ is 1000 genomes/LN. CFU limit of detection is 20/LN. Number of macaques and lymph nodes at each time point is in Table 1.

Not all thoracic LNs in an individual macaque become infected with Mtb (Fig 3A, S5 Fig). A significant proportion of thoracic LNs in individual macaques were CFU- (cynomolgus: 50–81%, rhesus: 26–40%, depending on time of necropsy) as supported by our PET CT data (Fig 1C). Although most CEQ+ LNs were also CFU+ (Fig 3B, pink bars), there were also CEQ+ LNs that were CFU- (Fig 3B, purple bars), suggesting that these LNs were able to completely sterilize the infection. Alternatively, it is possible that such LNs never have contained viable Mtb but we detected ‘free-floating’ Mtb genomes that were trapped by the LN; our limit of detection is 1000 CEQ per whole LN, so free floating DNA would have to be at reasonably high levels. These CFU-CEQ+ (purple bars) LNs were more prevalent in cynomolgus than rhesus macaques (Fig 3B). There were also LNs without detectable Mtb genomes but contained culturable Mtb (CEQ-CFU+) (Fig 3B, yellow bars) which likely represent samples where the number of Mtb genomes were below the limit of our qPCR assay. Overall, these data indicate that although it is possible for immune responses in LNs to kill Mtb, viable bacteria can remain in LNs for extended periods of time, suggesting these organs represent sites of long-term bacterial persistence.

### Low Mtb burden and better killing in peripheral lymph nodes

**Fig 4:**
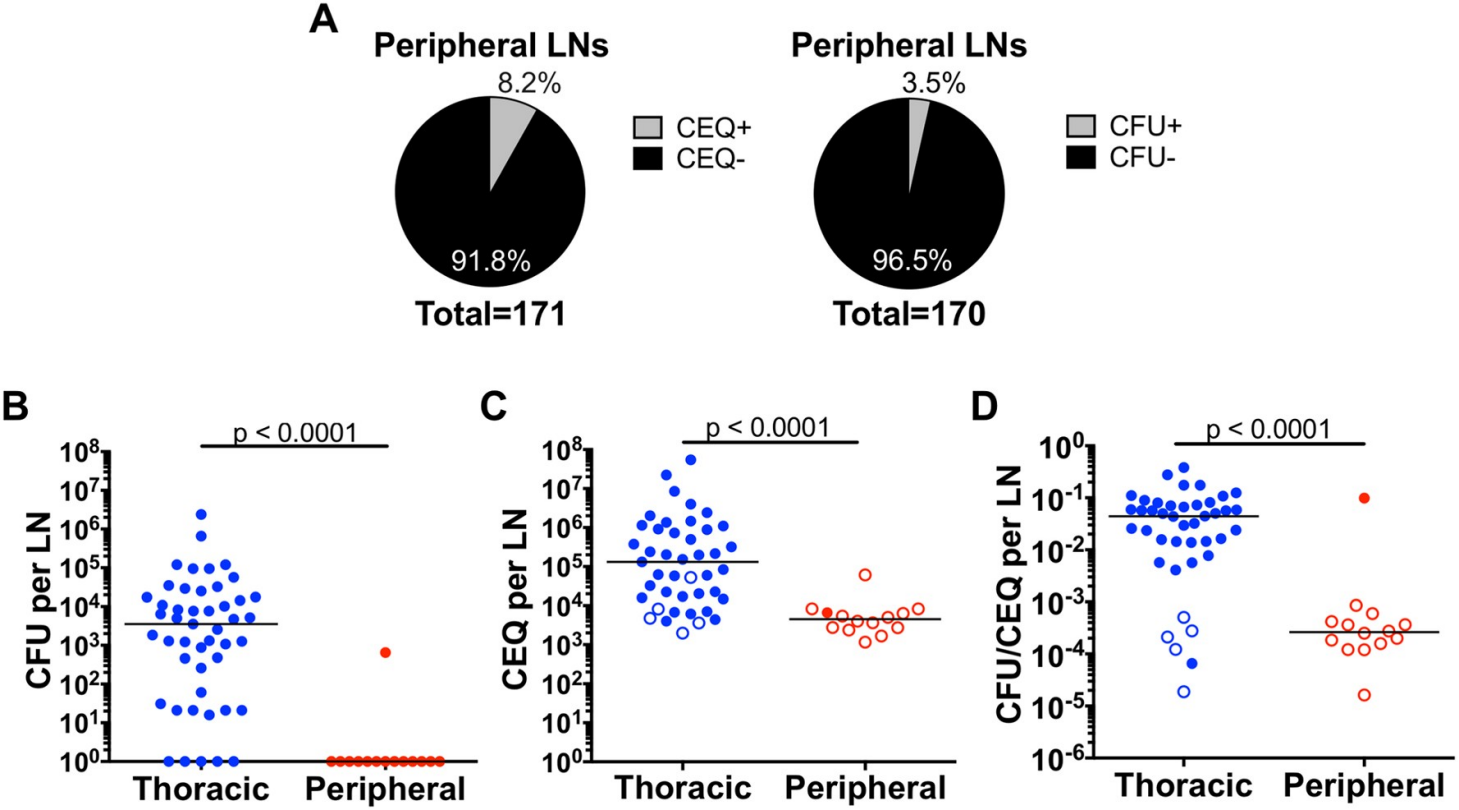
Most peripheral lymph nodes that had detectable Mtb genome were sterile. A. The majority of peripheral lymph nodes assayed were CEQ- and CFU- (sterile). B, C. Live Mtb burden (CFU)(B) and total (live+dead, CEQ) Mtb burden (C) are significantly lower in peripheral lymph nodes than in thoracic lymph nodes. D. CFU/CEQ for thoracic and peripheral LNs. Killing capacity of peripheral lymph nodes is significantly higher (lower CFU/CEQ) compared to thoracic lymph nodes. These are data from 10 monkeys that had CEQ in both thoracic and peripheral lymph nodes. Each data point is one lymph node. Open symbols are sterile (CFU-) lymph nodes. The CFU was transformed by adding 1 to reflect sterile but CEQ+ LNs. Statistics are Mann-Whitney.

Peripheral LNs, such as axillary and inguinal LNs, do not directly drain the lungs but may offer insight into extrapulmonary dissemination and immunity. To quantify mycobacterial dynamics in these organs, we sampled peripheral LNs for live Mtb and Mtb genomes from all macaques in this study and compared them to thoracic LNs. We found that only 8.2% (14 of 171) of the peripheral LNs we examined yielded Mtb genomes, and only 3.5% (6 of 171) contained viable Mtb, and these were at quite low levels (Fig 4A); this is not surprising since Mtb infection is generally confined to the thoracic cavity. Extrapulmonary disease, if present, is most frequently found in the liver, and occasionally the spleen, as noted in our previous publications [27, 28, 35]. We compared the live and total (live+dead) bacterial burden, as well as killing capacity of LNs from ten macaques (n = 5 cynomolgus, n = 5 rhesus) that had CEQ from both thoracic and peripheral LNs. Peripheral LNs had significantly lower levels of CFU compared to thoracic LNs (Fig 4B). Because the CFU levels were so low, the majority of the CFU+ LNs were outside the CEQ assay’s limit of detection. Here (Fig 4), we are only showing CFU, CEQ and CFU/CEQ data from CEQ+ LNs. The number of Mtb genomes recovered from peripheral LNs was significantly lower (29-fold, p<0.0001) compared to thoracic LNs (Fig 4C). Since we found significantly more live Mtb in thoracic LNs and most of the peripheral LNs were sterile (Fig 4B), the killing capacity of peripheral LNs was significantly higher (168-fold, p<0.0001) than thoracic LNs (Fig 4D). Our data suggest that Mtb infection of peripheral LNs can occur but is infrequent, and when it does occur, growth is to lower levels (CEQ) and these LNs are more likely to kill Mtb than thoracic LNs. However, trafficking of dead Mtb or Mtb genomes to peripheral LNs could also occur and should not be discounted.

### Lymph node effacement is associated with higher bacterial burden

**Fig 5:**
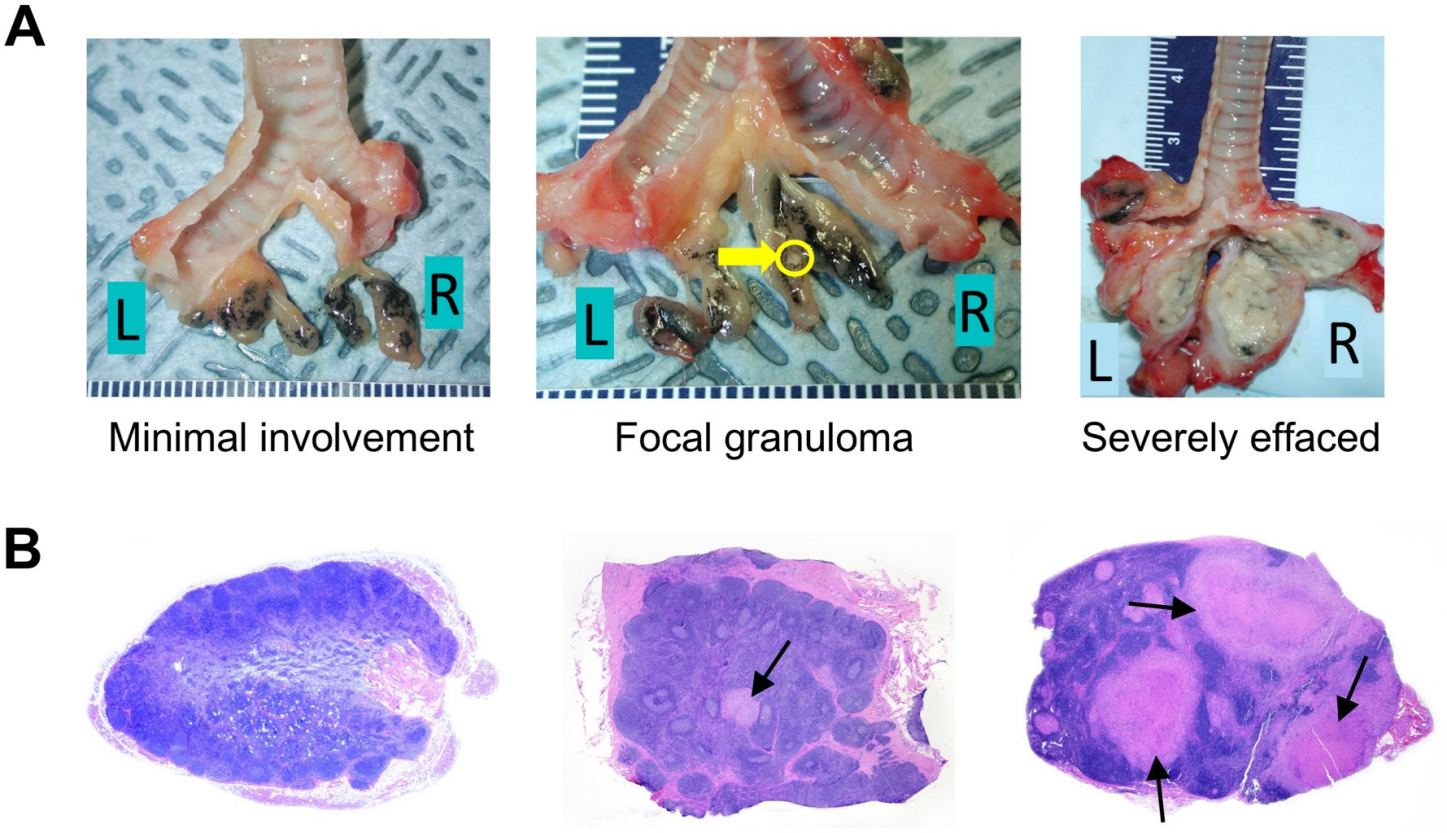
Mtb infection results in granuloma formation and in some instances lymph node effacement. A. Examples of gross pathology of thoracic lymph nodes from cynomolgus macaques that are minimally involved (left), with focal granuloma (middle) and severely effaced (right). The yellow arrow is pointing to a granuloma. B. Examples of microscopic histopathology of cynomolgus macaque lymph nodes that are not involved (left), with focal granuloma (middle) and severely effaced (right). The arrows are pointing to granulomas.

**Fig 6:**
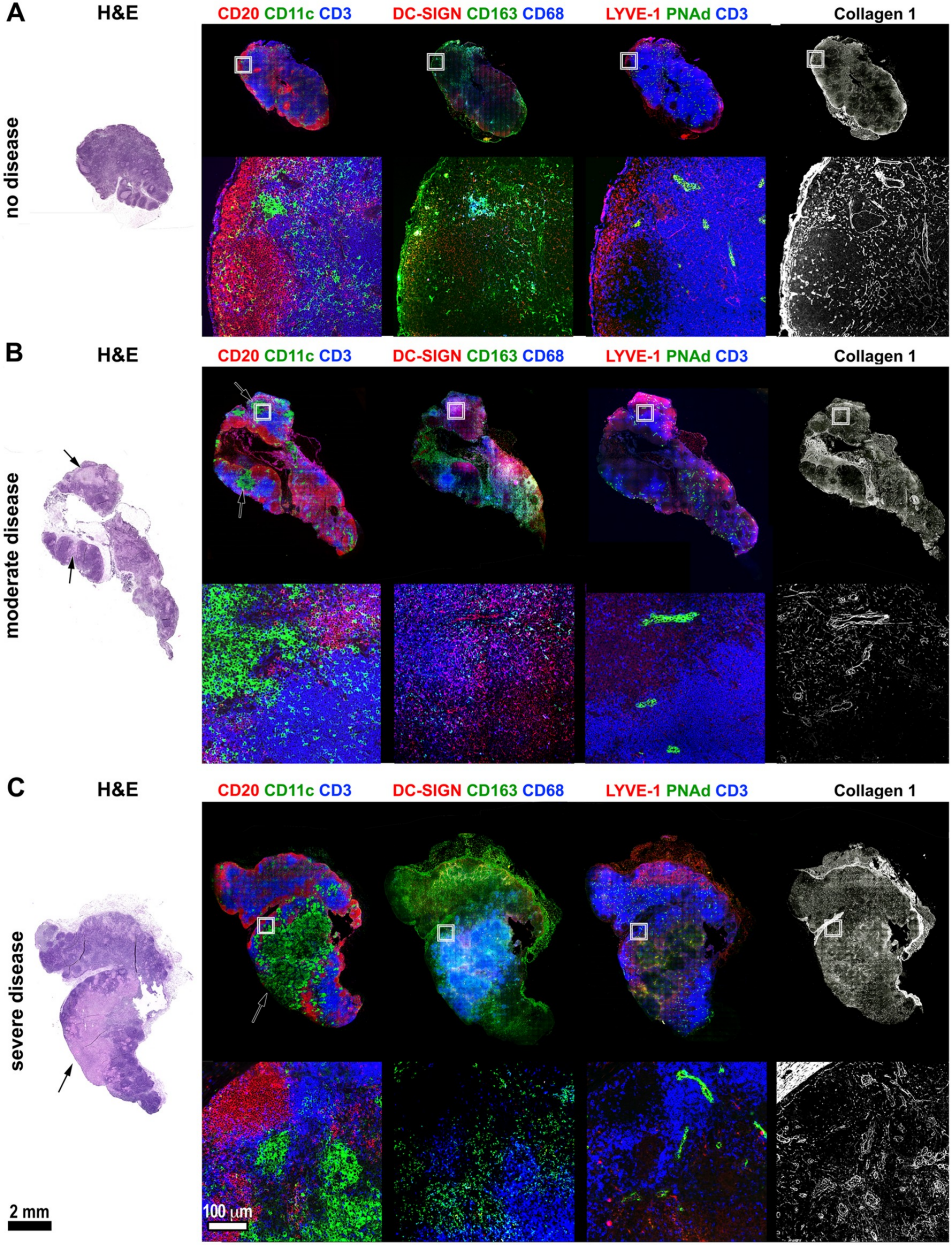
Histologic and immunohistochemical characterization of Mtb-infected macaque lymph nodes with varying levels of disease. FFPE tissue sections from Mtb-infected macaques were stained with hematoxylin and eosin (H&E) to show the tissue morphology and immunohistochemistry was performed on serial sections to identify the lymph node’s cellular, vascular, and structural elements. The box in the full-lymph node image indicates the region for the magnified panels (below) A. Lymph node showing no histologic evidence of disease and normal lymph node architecture. B. Lymph node demonstrating histologically-moderate disease where focal granulomas are present in T cell regions but not yet distorting the overall nodal architecture. Arrow indicates a granuloma. C. Severe lymph node disease showing large-scale disruption of the normal nodal structure in the vicinity of large coalescing granulomas. Arrow indicates a granuloma. Black scale bar (lower left) for the full-scale lymph nodes represents 2 mm. White scale bar (lower left, second column) for magnified image fields, represents 100 μm.

Mtb infection leads to granuloma formation in thoracic LNs and these granulomas can be focal or coalescing lesions that grow in size and efface the LN (Fig 5A and 5B). We previously reported that bacilli from multiple granulomas can seed a single LN [55, 61], and this is supported by histologic evidence where multiple independent granulomas are observed in a single LN (Fig 5B). A LN’s function is tightly linked to its physical structure and organization, and granulomas may physically disrupt LN architecture and impair their ability to function (ie. lymphatic filtration and immune cell trafficking to, from and within the LN). We performed immunohistochemistry (IHC) on cynomolgus macaque thoracic LNs with or without granulomas to investigate how granulomas influence the localization of cells, blood vessels, and the conduit systems that are important for normal LN function (Fig 6). We selected lymphocyte markers CD3 (T cells) and CD20 (B cells). Myeloid cell markers in lymph nodes are complex because dendritic cells (DCs) and epithelioid macrophages express both CD11c and DC-SIGN but can be distinguished by their different sizes and morphologies, while macrophages also express CD68 and can express CD163. For structural studies, we used markers defining vessels and conduit systems in LNs including LYVE-1 for lymphatic vessels, PNAd for high endothelial vessels (HEV), and collagen 1 (col1) for conduit systems. We focused on LNs from cynomolgus macaques to capture LNs from the full range of infection outcomes. Uninfected thoracic LN organization is consistent with typical LN architecture with CD3+ T cells and CD11c+ DCs being abundandant in paracortical regions, CD20+ B cell-rich germinal centers at the periphery, and CD68+ and CD163+ macrophages present in the subcapsular space and medullary region (Fig 6A). Mesh-like LYVE-1+ lymphatic vessels were present in the central LN region while PNAd+ HEV are distributed throughout the paracortex, and col1+ conduits regularly-spaced through the T cell regions.

Mtb-infected thoracic LNs can contain multiple focal non-necrotizing granulomas or large coalescing granulomas that disrupt the LN architecture. We found that even small granulomas (Fig 6B) had large clusters of CD11c+CD68+ macrophages that appeared to push T cells out of these regions and impinged on germinal centers, but also disrupted nearby HEV and lymphatic vessels. Moreover, granulomas and adjacent areas had disorganized col1 staining instead of the uniformly-distributed staining associated with the nomal conduit network found in uninvolved areas. Large granulomas caused extensive remodeling of LN structure. Effaced LNs (Fig 6C) had coalescing necrotic granulomas with poorly-circumscribed margins and large numbers of CD11c+CD68+ macrophages, and these granulomas displaced T cell- and DC-rich zones in the LN paracortex, destroyed B cell-rich germinal centers, and eliminated the normal vascular elements in their vicinity. The granulomas in these LNs stained positively for col1, but as with less involved LNs, the staining is disorganized and lacks cohesive conduit-like organization. Interestingly, LN granulomas lack several features that are present in lung granulomas. Although these granulomas are present in B cell and T cell-rich organs, they appear to lack granuloma-adjacent B cell-rich tertiary lymphoid structures [62, 63], and distinct lymphocyte cuff regions. These observations suggest LN granulomas have large populations of potential host cells, but have structural differences that may impair the ability to control bacterial replication. Moreover, the process of granuloma formation in thoracic LNs can destroy important aspects of LN structure that contribute to T cell and B cell priming and may affect overall anti-mycobacterial immunity.

**Fig 7:**
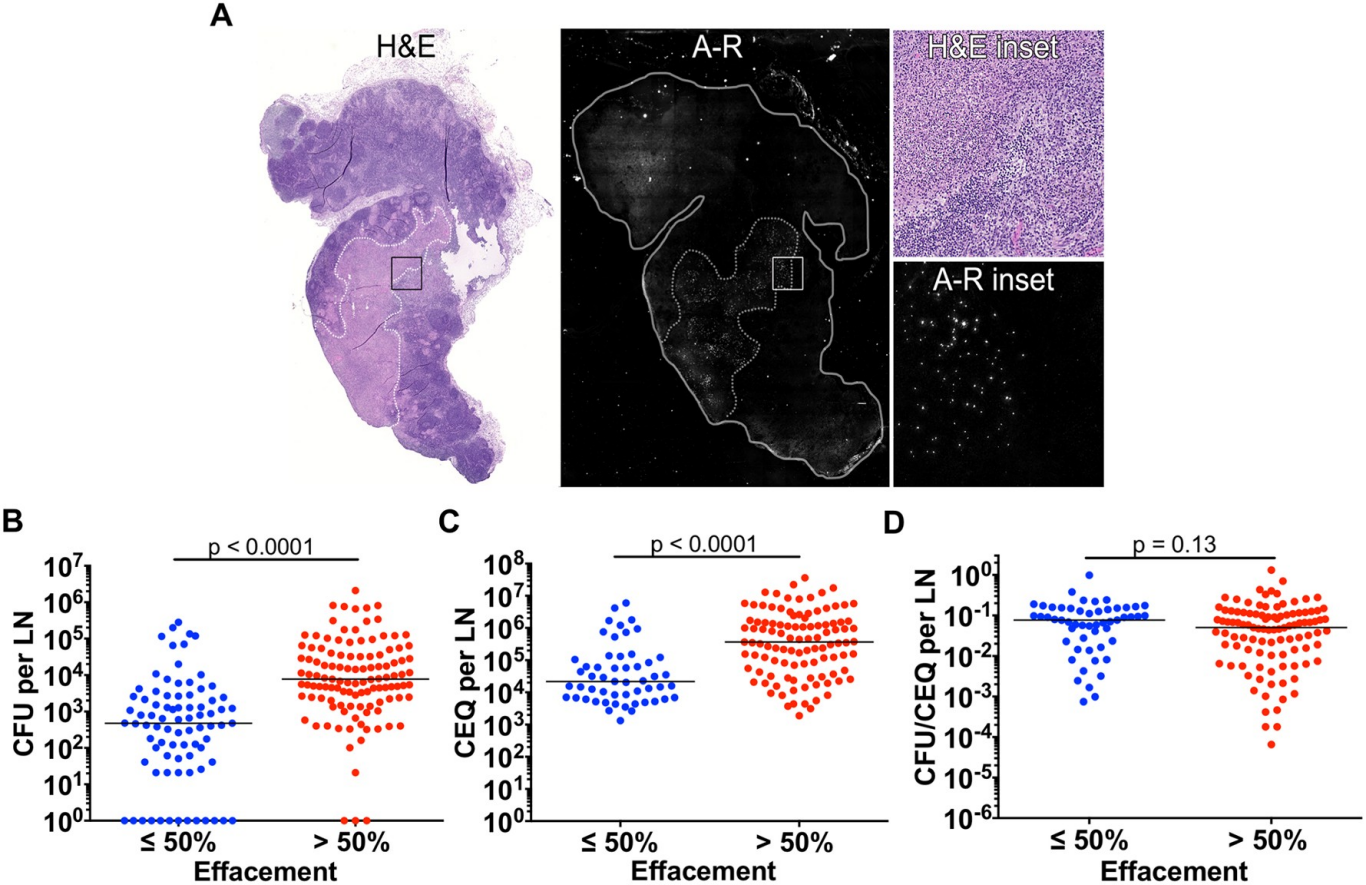
Lymph node effacement promotes Mtb growth. A. H&E and Auramine-rhodamine (A-R) staining of a severely-effaced thoracic lymph node (previously depicted in Fig 6C). The location of a large granuloma, indicated by white and grey dashed lines in the H&E and A-R panels, respectively, corresponds with substantial numbers of A-R-stained Mtb. Inset regions (black and white boxes in the H&E and A-R panels, respectively) show the interface between granulomatous and non-granulomatous lymph node regions. B, C. CFU (B) and CEQ (C) of thoracic lymph nodes with **≤**50% or >50% effacement (effacement determined by H&E section). D. Mtb killing capacity (CFU/CEQ) of lymph nodes are not affected by the degree of effacement. Each data point is one lymph node. The CFU was transformed by adding 1 to reflect sterile but CEQ+ lymph nodes. Statistical test is Mann-Whitney.

We hypothesized that effaced LNs (i.e. extensive necrosis) would be an excellent site for Mtb growth so we used auramine-rhodamine (A-R) staining to identify where bacteria localize in LNs. We found that Mtb, visualized as numerous small puncta in A-R stained sections (Fig 7A, A-R inset), were abundant in granulomatous regions but absent from granuloma-free regions suggesting granulomas are foci of bacterial persistence and replications in LNs. When LNs with different levels of effacement were compared for CFU and CEQ, LNs with >50% effacement had significantly higher CFU and CEQ than those with ≤50% effacement (Fig 7B and 7C) but there was no difference in the killing capacity of LNs with differing degrees of effacement (Fig 7D). The increase in both CEQ and CFU, and lack of killing, suggests that the macrophage populations and necrotic regions associated with effacement are conducive to Mtb persistence and replication.

### Bacterial burden is associated with decreased capacity to induce cytokine production in LNs with granulomas

Cytokine producing cells play important roles in the control of Mtb infection [64]. Although LNs are sites of T cell priming for control of infection in lung granulomas, they also must generate functional cells to control infection within the LNs. We evaluated cytokine expression using multiparameter flow cytometry in thoracic LN cells from 24 cynomolgus macaques (S2 Table) included as controls in other studies. We investigated T cell, B cell, and macrophage expression of proinflammatory cytokines: Th1(IFNγ, IL-2, TNF), Th17 (IL-17) and anti-inflamtory cytokine IL-10 following stimulation with peptide pools from Mtb antigens ESAT-6 and CFP-10. LNs with granulomas (identified either by gross pathology or histopathology) were evaluated for this analysis. Uninfected LNs (no granuloma established and no live Mtb) had similar cytokine profile as that of LNs with granuloma that cleared Mtb, however uninfected LNs had significantly higher proportions of CD3 T cells (p = 0.0009) than LNs with granulomas. The number of LNs for each analysis varied by the panel used in that particular study (S3 Table).

**Fig 8:**
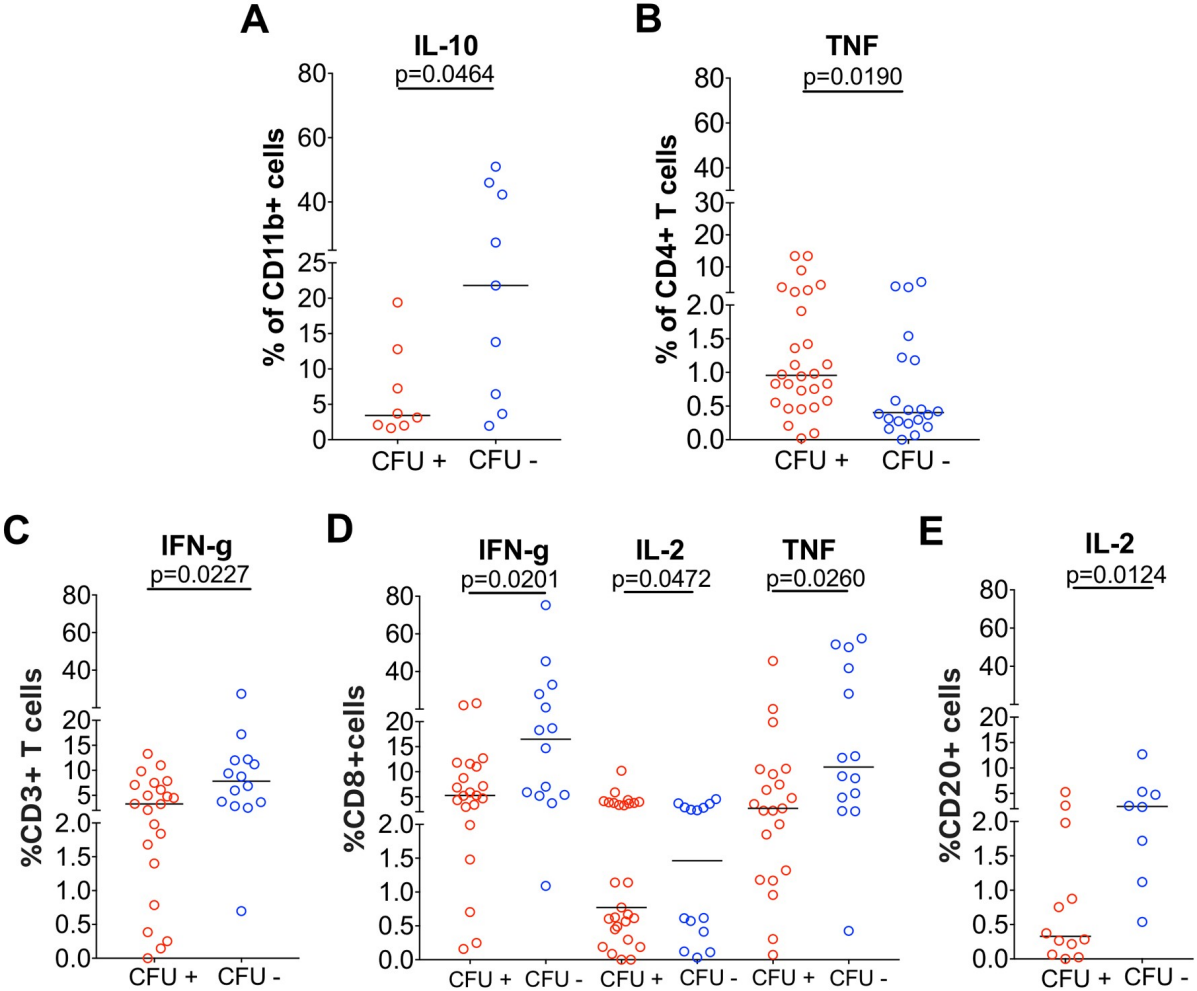
Immune response in thoracic LNs of cynomolgus macaques with granulomas. Cytokine production in thoracic LN with granuloma in response to ESAT6+CFP10 (A-B) or PDBu and ionomycin (C-E) between LNs with bacterial burden (CFU+, red) and those that cleared (CFU-, blue). A. Frequency of CD11b+ cells producing IL-10 (n = 10 macaques; 27 LNs); B. Frequency of CD4+ T cells producing TNF (n = 24 macaques; 48 LNs). C. Frequency of CD3+ cells producing IFNγ(n = 12 macaques; 35 LNs); D. Frequency of CD8+ cells producing IFNγ(n = 12 macaques, 34 LNs) IL-2 (n = 10 macaques; 28 LNs) and TNF (n = 12 macaques; 34 LNs); E. Frequency of CD20+ cells producing IL-2 (n = 11 macaques; 20 LNs). Each symbol represents a LN. Statistical test is Mann-Whitney.

First, we compared cytokine reponses between thoracic LNs with granuloma that had live Mtb (CFU+) and those that cleared Mtb (CFU-). As seen in lung granulomas [65], we found that LN granulomas represent a multi-cytokine environment with the presence of both pro and anti inflammatory cytokines produced by a variety of cells within a LN. Cytokine responses largely overlapped between CFU+ LNs and CFU- LNs (S3 Table). Nevertheless, CFU- LNs with granulomas had significantly higher proportions of CD11b+ cells producing IL-10 (Fig 8A) when compared with CFU+ LNs with granulomas, while CD4+ T cells producing TNF (Fig 8B) were significantly higher in CFU+ LNs (S3 Table Panel A). With the exception of TNF response, there was no significant difference in cytokine producing T, B and CD11b+ cells in CFU+ and CFU- LNs (S3 Table Panel A) in response to Mtb antigens ESAT-6 and CFP-10. Secondly, we questioned whether there was an association between cytokine response and bacterial burden. We found a significant negative correlation between bacterial burden and CD11b+ cells producing IL-10 (Spearman’s ρ -0.5046, p = 0.0389) (S3 Table Panel C), and a positive correlation with bacterial burden and CD4 T cells producing TNF (Spearman’s ρ 0.2892, p = 0.0462) (S3 Table Panel C). These data suggest that IL-10 response from macrophages is associated with bacterial clearance while CD4+ T cell TNF response could be attributed to onging Mtb replication.

Next, we investigated whether Mtb burden alters the overall capacity of cells in thoracic LNs with granuloma to produce cytokines by stimulating them non-specifically with PDBu and ionomycin. Overall, sterile (CFU-) LNs have a trend towards cells producing higher frequency of cytokines compared to CFU+ LNs. Of note, LNs with granulomas that are sterile had significantly higher frequencies of CD3+ T cells producing IFNγ (Fig 8C); CD8+ cytotoxic T cells producing IFNγ, IL-2 or TNF (Fig 8D) and CD20+ B cells producing IL-2 (Fig 8E) than CFU+ LNs. In addition, we also observed a higher proportion of CD11b+ cells producing IFNγ and IL-10 (S3 Table Panel B) in LNs without bacterial burden under the same conditions, even though PDBu-ionomycin does not induce these cytokine production in CD11b+cells. These data suggest that LNs that have sterilized the infection have a higher capacity to produce both pro- and anti-inflammatory cytokines and this may have contributed to its ability to eliminate Mtb.

We extended our investigation to understand whether there are differences in Mtb-specific responses between thoracic and peripheral LNs to determine whether these responses could contribute to protection in the periphery. For this analysis, 14 peripheral LNs and 27 thoracic LNs from 7 animals were evaluated (S4 Table). None of the peripheral LNs had any granuloma or grew Mtb from culture, while 14 of thoracic LNs had granulomas and 12 thoracic LNs grew Mtb. Peripheral LNs had significantly higher proportion of CD3+ (S6A Fig) and CD4+ T cells (S6B Fig) than thoracic LNs (S4 Table). Irrespective of bacterial burden or granuloma presence, the proliferative capacity of T cells, as measured by Ki67+ CD3+ T cells, especially Ki67+ CD8+ cytotoxic T cells (S4 Table, S6C Fig), was significantly higher in thoracic LNs than in peripheral LNs, suggesting that Mtb induced proliferation might occur in the thoracic LNs. There was no difference in T cell cytokine response or the capacity to induce cytokines by T cells as determined by PDBu and ionomycin stimulation between thoracic and peripheral LNs (S4 Table).

Finally, since we observed that the thoracic LNs with higher bacterial burden had decreased capacity to produce certain cytokines when compared to those with no bacterial burden, we examined whether the degree of LN effacement altered immune cell function in a subset of thoracic LNs where data for degree of effacement (n = 55) were available. There was no difference in cytokine responses to Mtb specific antigens, capacity to produce cytokines in LNs without granulomas, ≤50% effaced LNs, and >50% effaced LNs (S5 Table) suggesting that LN effacement does not alter the functional capacity of LN cells.

### Short course drug treatment is more effective in lung granulomas than in thoracic lymph nodes

Since thoracic LNs are generally poor at killing Mtb, we examined whether there was a relationship between LN involvement and extrapulmonary disease. Greater lymph node involvement, as assessed by gross pathology scoring (which accounts for number of LNs with granulomas, size of LN, and effacement) was associated with more extrapulmonary disease in rhesus (F test, R^2^ = 0.588, p < 0.0001) but not in cynomolgus macaques (S7A Fig). Extrapulmonary score was not related to total LN bacterial burden in either macaque species (S7B Fig). We also examined data from a previous study [57] to determine whether Mtb in thoracic LNs was less effectively killed during a short-course (2 months) linezolid (LZD) treatment relative to lung granulomas. Lung granulomas had a higher proportion of sterility (75% LZD-treated vs. 21.8% control) after drug treatment compared to thoracic LNs (16.7% LZD-treated vs. 2.5% control). The reduction in bacterial burden in LNs (S8A Fig) was lower than the reduction of bacterial burden in lung granulomas (S8B Fig) of LZD-treated macaques (55-fold vs. 181-fold, respectively) compared to untreated control macaques. These data suggest that after 2 months of LZD treatment, Mtb was killed less effectively in thoracic LNs compared to lung granulomas, supporting that LNs are bacterial reservoirs and potential sites of reactivation or relapse. This is consistent with our previously published observation in which short course isoniazid and rifampin treatment for 2 months was more effective in reducing bacterial burden in lung granulomas than in thoracic LN during active disease treatment [66].

## Discussion

Traditionally, lung infection has been the primary focus of TB research and treatment, despite evidence that Mtb also infects and persists in LNs. There are unanswered questions regarding the importance of LN infection in the pathogenesis of TB including bacterial replication or killing, and the antimicrobial immune responses in LNs. Here we focused on LN infection in cynomolgus and rhesus macaques, two closely-related nonhuman primate species that replicate the pathology of human TB [27–29]. We present data supporting that LNs can become inflamed (FDG-avid) as early as 2 weeks post infection. Starting at 4 weeks post-infection, FDG activity correlated with the presence of live bacteria in LNs at necropsy. Although thoracic LNs in cynomolgus macaques initiate killing of Mtb by 11 weeks post-infection, with substantially more killing at later time points, LNs from rhesus macaques have impaired killing capacity, likely contributing to the increased susceptibility of this species. We also found that granulomas substantially disrupt the structure of LNs, and can completely efface the organ, which is associated with increased growth of Mtb. Overall, our data support that thoracic LNs are a site of prolonged bacterial persistence, sometimes at quite high levels.

Previous work has demonstrated that LNs are important in reactivation TB [11, 32–34] but little is known about the interplay of Mtb infection and host responses in LNs. Large human studies by Poulsen in the 1930s and 1940s showed that early after Mtb infection, there was often an initial fever that lasted 2–3 weeks [67, 68]. These subjects presented with enlarged “hilar shadows” by x-ray, assumed to be lymphadenopathy. A large number of subjects also presented with hilar adenitis when they first became tuberculin skin test positive which they ascertained to be ~40 days after infection. To investigate the dynamics of Mtb infection in LNs of macaques, we used PET CT to monitor LNs over the course of infection. In support of Poulsen’s early data, we found increased metabolic activity (FDG uptake) in thoracic LNs as early as 2 weeks post-infection and more LNs became FDG-avid by 4–6 weeks. This increase in FDG uptake is associated with Mtb infection in that nearly all FDG+ LNS are also CFU+ at necropsy, and is likely due to increased metabolic activity of cells during priming of the adaptive immune response as well as the host cells reacting to the presence of Mtb bacilli. However, not all LNs in an individual macaque became infected, and those that were infected could wax and wane in FDG avidity over the course of infection.

Rhesus and cynomolgus macaques are closely related species used in TB research, and we recently performed quantitative analyses of similarities and differences between Mtb infection outcomes in these species [35]. Cynomolgus macaques develop the full range of infection outcomes seen in humans, from clinically latent to severe active TB. In contrast, rhesus macaques are more susceptible, with nearly all animals developing active and often severe TB within several months of experimental low dose infection. One striking feature of TB in rhesus macaques is the often substantial involvement of thoracic LNs with high levels of necrosis; in some cases a LN can grow massively and impinge on the airways causing lobe collapse or erode into a bronchus leading to further dissemination. Certainly, substantial LN involvement can be seen in some cynomolgus macaques, but in general the extent of LN disease is lower in this species [35]. A limitation of comparing these models is that due to the susceptible nature of rhesus macaques, essentially none of these animals present with long term controlled (latent) infection following low dose challenge with virulent Mtb and nearly all succumb before 24 weeks of infection. Thus, LN samples from long term (>26 weeks post-infection) rhesus macaques for comparison with similar time points in cynomolgus macaques are not available.

Here, we show that Mtb grows to the same initial level (with a wide range) in thoracic LNs at 4 weeks post infection in both species, but rhesus lymph nodes were less successful at killing Mtb over time. Remarkably, at 16–29 weeks post-infection, the different disease states (controlling to active disease) of cynomolgus macaques were reflected in the capacities of their LNs to kill Mtb. This further supports our findings that the lungs and LNs are intricately linked during Mtb infection [35]. However, in general, LNs were less bactericidal than lung granulomas in both species [55]. This deficit in bacterial killing at the LN level in part explains the pronounced LN disease and increased susceptibility of rhesus macaques to Mtb infection [35]. Despite the limitation of CEQ as being simply an estimate of Mtb genomes, it is the only technology at this time that is available to estimate live + dead bacilli and actual killing of Mtb (together with CFU measurement) in vivo. Lung granulomas peak in bacterial burden early in infection with approximately 10^4^−10^5^ CFU per granuloma, followed by substantial killing of bacteria in most granulomas once the adaptive immune response is initiated [55]. In contrast, some thoracic LNs had 100-fold more bacteria than lung granulomas. In cynomolgus macaques, we show that even when granulomas are established, some LNs are able to clear Mtb and these LNs are associated with higher CD11b+ cells producing IL-10. LN bacterial burden was inversely correlated with CD11b+ cells producing the anti-inflammatory cytokine IL-10 and sterile LNs had significantly higher capacity to produce both pro- and anti- inflammatory cytokines, suggesting this multi-cytokine environment might contribute to bacterial containment in a subset of LNs, similar to our observations in lung TB granulomas [65]. We did not have sufficient samples to conduct a full kinetic analysis of the CD11b+IL-10+ population; this finding requires further study to understand its significance.

Somewhat surprisingly, we discovered Mtb genomes in peripheral LNs (i.e., axillary and inguinal) that do not drain the lungs, even though most of these LNs were sterile. Although our data suggest that occasionally peripheral LNs can be infected with small numbers of bacteria and are better at killing Mtb than thoracic LNs, we cannot exclude the possibility that genomes detected here were free-floating DNA trapped in these tissues. Histologic analysis did not reveal evidence of pathology in the CEQ+ peripheral LNs, even in the few that had viable Mtb. The difference in the cytokine responses and proliferative markers we observed between peripheral and thoracic LNs could be attributed to constant stimuli from Mtb at the thoracic LNs. The improved sterilizing capacity of peripheral LNs could also be due to the timing of infection in these LNs, which we could not ascertain in our study.

The structure and spatial organization of cells in LNs are critical for their function [69, 70], and may also help explain why LNs have limited abilities to kill Mtb. Even though the killing capacity and immune function of LNs with varying degrees of effacement are similar, the bacterial burden (CEQ and CFU) in >50% effaced LNs was 16-fold higher compared to ≤50% effaced LNs. Our data support that the more extensive the disruption in LN structure is, the more conducive the LN is for Mtb growth and this is likely due to a number of factors. First, the formation of a granuloma recruits a large number of macrophages that can serve as niches for Mtb growth. Moreover, as LN granulomas expand they push out T cells, disrupt B cell follicles, which could interfere in antibody production that could aid in controlling infection [71, 72], and damage LN-associated vasculature, which could potentially change drug availability. This disruption of LN architecture may also affect resident innate immune cells that have been shown to be spatially pre-positioned to provide a cascade of cytokines which promote macrophage antimicrobial resistance limiting pathogen dissemination [47]. In contrast to lung granulomas [62, 73, 74], LN granulomas do not have a well-defined lymphocytic cuff with tertiary lymphoid structures surrounding the epithelioid macrophage region. As the presence of these structures in lung granulomas has been associated with protection in pulmonary TB [63, 75, 76], we speculate that the absence of these structures results in poorly functional granulomas that have limited capacity to restrain or kill Mtb. These observations suggest that granulomas can potently influence aspects of LN architecture that facilitate T cell priming and systemic immunity, while providing an abundance of extra- and intracellular sites for mycobacterial replication.

Early autopsy studies support the idea that LNs serve as long-term reservoirs for Mtb. Viable Mtb bacilli were found in 9.4–27% of LNs from autopsies of patients without evidence of TB disease in lungs or anywhere in their bodies [77–79]. Mtb infection of LNs may also have implications in drug treatment. In our study, we found that a short-course LZD therapy was less effective in killing Mtb in LNs compared to Mtb in lung granulomas and this was also observed in short course isoniazid with rifampin [66]. Indeed, in a study that followed 113 patients that relapsed for 30 months, tuberculous lymphadenitis was found as a risk factor for relapse [80]. Of the 12 patients that had both pulmonary and LN TB, 9 (75%) patients had a recurrence exclusively in the LNs, while the remaining 3 patients had recurrence in both lungs and LNs [80]. However, only 1 patient was confirmed by bacterial culture and lymphadenopathy could also be caused by an impaired regulation of the immune system. In HIV+ patients with clinically latent TB, the presence of abnormal FDG uptake in mediastinal LNs was associated with patients showing subclinical TB disease and the likelihood of developing symptomatic TB disease during the 6 month follow up [11]. These studies suggest that Mtb can persist in LNs even after drug treatment, which we observed as well, and increased FDG activity in these tissues are associated with persisting or reactivating TB disease.

This study presents an in-depth investigation into bacterial dynamics and immunity in thoracic LNs during Mtb infection. Our data indicate that LNs can contain large numbers of bacteria and serve as long-term reservoirs of bacterial persistence. Thus, understanding how non-protective LN granulomas differ from protective lung granulomas may lead to strategies that improve TB treatment and outcomes. Moreover, our study identifies LNs and LN infection as important considerations for measuring vaccine and treatment efficacy.

## Materials and methods

### Animals

Cynomolgus (*Macaca fascicularis*) (n = 32) and rhesus macaques (*Macaca mulatta*) (n = 19) that served as controls (no vaccine or drug treatment) for other studies from 2011 to 2016 were selected for this study. These macaques were infected with a low dose (~1–28 CFU, median = 6 CFU) of Mtb strain Erdman using a bronchoscope. The animals used in this study are summarized in Table 1. The number of LNs examined per monkey ranged from 4–21, with the median being 12 per monkey. A detailed list of animals can be found in Supplementary Table 1; other data from some monkeys have been previously published in other studies as noted in that table. All procedures and protocols were approved by the University of Pittsburgh’s Institutional Animal Care and Use Committee.

**Table 1:** Animals used in the CFU, CEQ, and histological studies.

Cynomolgus Macaques				
Time post-infection	No. of animals	No. of thoracic LNs	No. of peripheral LNs	Total No.
4–6 weeks	8	61	32	93
11–14 weeks	9	70	34	104
16–29 weeks	9	57	12	69
34–54 weeks	6	49	23	72
Rhesus Macaques				
Time post-infection	No. of animals	No. of thoracic LNs	No. of peripheral LNs	Total No.
4 weeks	4	38	16	54
11–14 weeks	7	59	26	85
16–28 weeks	8	79	28	107

### Ethics statement

All experimental manipulations, protocols, and care of the animals were approved by the University of Pittsburgh School of Medicine Institutional Animal Care and Use Committee (IACUC). The protocol assurance number for our IACUC is A3187-01. Our specific protocol approval numbers for this project are: 13122856, 15066174, 12080653, 15126588, 11110045, 12060181, 14023305, 1105870, 11090030, 15015299, 12090832, 15066174, 1003622, 1105870. The IACUC adheres to national guidelines established in the Animal Welfare Act (7 U.S.C. Sections 2131–2159) and the Guide for the Care and Use of Laboratory Animals (8^th^ Edition) as mandated by the U.S. Public Health Service Policy.

All macaques used in this study were housed at the University of Pittsburgh in rooms with autonomously controlled temperature, humidity, and lighting. Animals were singly housed in caging at least 2 square meters apart that allowed visual and tactile contact with neighboring conspecifics. The macaques were fed twice daily with biscuits formulated for nonhuman primates, supplemented at least 4 days/week with large pieces of fresh fruits or vegetables. Animals had access to water *ad libitem*. Because our macaques were singly housed due to the infectious nature of these studies, an enhanced enrichment plan was designed and overseen by our nonhuman primate enrichment specialist. This plan has three components. First, species-specific behaviors are encouraged. All animals have access to toys and other manipulata, some of which will be filled with food treats (e.g. frozen fruit, peanut butter, etc.). These are rotated on a regular basis. Puzzle feeders foraging boards, and cardboard tubes containing small food items also are placed in the cage to stimulate foraging behaviors. Adjustable mirrors accessible to the animals stimulate interaction between animals. Second, routine interaction between humans and macaques are encouraged. These interactions occur daily and consist mainly of small food objects offered as enrichment and adhere to established safety protocols. Animal caretakers are encouraged to interact with the animals (by talking or with facial expressions) while performing tasks in the housing area. Routine procedures (e.g. feeding, cage cleaning, etc) are done on a strict schedule to allow the animals to acclimate to a routine daily schedule. Third, all macaques are provided with a variety of visual and auditory stimulation. Housing areas contain either radios or TV/video equipment that play cartoons or other formats designed for children for at least 3 hours each day. The videos and radios are rotated between animal rooms so that the same enrichment is not played repetitively for the same group of animals.

All animals are checked at least twice daily to assess appetite, attitude, activity level, hydration status, etc. Following *M*. *tuberculosis* infection, the animals are monitored closely for evidence of disease (e.g., anorexia, weight loss, tachypnea, dyspnea, coughing). Physical exams, including weights, are performed on a regular basis. Animals are sedated prior to all veterinary procedures (e.g. blood draws, etc.) using ketamine or other approved drugs. Regular PET/CT imaging is conducted on most of our macaques following infection and has proved very useful for monitoring disease progression. Our veterinary technicians monitor animals especially closely for any signs of pain or distress. If any are noted, appropriate supportive care (e.g. dietary supplementation, rehydration) and clinical treatments (analgesics) are given. Any animal considered to have advanced disease or intractable pain or distress from any cause is sedated with ketamine and then humanely euthanatized using sodium pentobarbital.

### FDG PET-CT imaging

Serial 2-deoxy-2-[^18^F]-D-deoxyglucose (FDG) positron emission tomography (PET) with computed tomography (CT) imaging was performed in a biosafety level 3 facility as described previously [56]. Lymph nodes were identified by our analyst (P. Maiello) and metabolic activity (FDG avidity) was measured [81]. Lymph nodes that were seen on scan had a maximum SUV (standard uptake value) greater than or equal to 2.3 SUVR (maximum standard uptake value ratio normalized to muscle to reduce variability between scans). Despite the fact that we can see LNs with SUV ≥ 2.3 on the scans, we consider LNs with SUVR of ≥ 5 to be “hot.” Serial scans were performed ranging from 1 week to 54 weeks post-infection. The majority of the shorter term infection macaques (necropsied 14 weeks post-infection or earlier) were scanned every 2 weeks until necropsy. The longer term infection macaques (necropsied 15 weeks post-infection or longer) were scanned every 2 weeks until 8 weeks post-infection and every 4 weeks thereafter. Some of the latent monkeys (34–54 weeks) were only scanned immediately prior to necropsy.

### Necropsy

Necropsy procedures were as previously described [31]. Briefly, one to three days prior to necropsy, macaques were imaged by FDG-PET/CT to ascertain which LNs were metabolically active and to measure their FDG avidity. Individual lung granulomas, thoracic, axillary and inguinal LNs were excised and cut into 2 sections. One section was homogenized into single cell suspension in PBS for immunology and aliquots made for both plating on 7H11 agar plates to obtain colony forming units (CFU) and DNA extraction for qPCR. The other section was placed in 10% normal buffered formalin and paraffin embedded for histologic examination. Bacterial burden (CFU) per LN was determined by accounting for the amount of sample plated compared to the entire LN sample. The LN necropsy score was determined based on the number of LNs with granulomas, the size of the lymph nodes and the degree of LN effacement. The extrapulmonary score was determined based on the presence, relative frequency and size of granulomas in other areas of the body (eg. diaphragm, liver, spleen, other abdominal viscera) and the number of extrapulmonary sites (excluding lymph nodes) that had bacterial burden. Our necropsy scoring system is available in [35].

### Flow cytometry

Intracellular cytokine staining was performed on a random sampling of LNs with and without granuloma from each animal (n = ~4/animal). A total of 168 LNs (with and without granuloma) from 24 cynomolgus macaques that were part of other studies in our lab were included in this analysis and processed as previously described [65]. Single cell suspension of 96 LNs were stimulated with peptide pools of Mtb specific antigens ESAT-6 and CFP-10 (10 μg/ml of every peptide) in the presence of Brefeldin A (Golgiplug: BD biosciences) for 3.5 hours at 37°C with 5% CO_2_ [65]. Positive control (n = 72 LNs) included stimulation with phorbol dibutyrate (PDBu) and ionomycin [31]. An unstimulated control was included whenever additional cells were available. The cells were then stained for viability (Invitrogen), surface and intracellular cytokine markers according to standardized protocols. Flow cytometry panel for cell surface markers for T cells included CD3 (clone SP34-2; BD Pharmingen), CD4 (Clone L200, BD Horizon) and CD8 (clone SK1: BD biosciences). In addition, the B cell marker CD20 (clone 2H7; eBioscience) and myeloid cell marker CD11b (clone Mac-1, BD Pharmingen) were included as additional markers in certain samples. Intracellular cytokine staining panel included pro-inflammatory cytokines: Th1 [IFN-γ (clone B27), IL-2 (clone: MQ1-17H12), TNF (clone: MAB11)], Th17 [IL-17 (clone eBio64CAP17)] and the anti-inflammatory cytokine IL-10 (clone JES3-9D7) markers. In addition, T cell proliferation marker Ki67 [clone B56] was included in the panel for a subset of samples. Data acquisition was performed using an LSR II (BD) and analyzed using FlowJo Software v.9.7 (Treestar Inc, Ashland, OR). A detailed list of macaques included in the analysis can be found in S2 Table.

### Histology

Histological examination was performed by an experienced veterinary pathologist (E. Klein) as previously described [31]. Tissue samples were cut (4-6mm) and stained with hematoxylin and eosin. Characteristics of granulomas, such as, size, type (caseous, non-necrotizing, suppurative, or mixed), distribution pattern (focal, multifocal, coalescing, focally extensive and locally invasive), and cellular composition were noted.

### Immunohistochemistry

Immunohistochemistry was performed as previously indicated [73, 82] on formalin-fixed paraffin-embedded (FFPE) LNs obtained at necropsy. Briefly, sections were deparaffinized and antigen retrieval was performed using a Retriever (Electron Microscopy Services, Hatfield, PA) in Tris-EDTA-Tween-80 buffer 73. Sections were stained for Tcells/B cells/dendritic cells (polyclonal rabbit anti-CD3, Dako, Santa Clara, CA; polyclonal rabbit anti-CD20, Thermo Fisher Scientific, Pittsburgh, PA; mouse-anti-CD11c, Leica Microsystems, Buffalo Grove, IL), macrophage subsets (mouse anti-CD68, Thermo Fisher; rabbit anti-DC-SIGN, ProSci Inc, Poway, CA; mouse anti-CD163, Thermo Fisher), LN vascular and structural aspects (Goat anti-LYVE-1, R&D Systems, Minneapolis, MN; rat-anti PNAd, BioLegend, San Diego, CA), and LN conduit systems (visualized by staining for rabbit anti-collagen 1 [Abcam, Cambridge, MA]). Primary antibodies were visualized with species- and isotype-specific secondary antibodies purchased from Jackson ImmunoResearch (West Grove, PA). Auramine rhodamine was performed as previously indicated [73] using reagents from BD Biosciences (San Jose, CA). Images were acquired at 20x magnification with a Nikon e1000 widefield microscope (Nikon, Melville, NY) with Nikon Elements.

### Mtb genome isolation and quantification

DNA extraction and qPCR was performed with modifications as described previously [55]. Briefly, frozen aliquots were thawed and volumes recorded throughout the extraction process. Samples were transferred to tubes containing 150 μl of 0.1mm zirconia-silica beads (Biospec Products) before adding 600μl of Tris-EDTA buffer, pH 8.0. Three hundred microliters of 70°C phenol/chloroform/isoamyl alcohol (25:24:1, Sigma-Aldrich) were subsequently added and the samples incubated at room temperature for 10 minutes. The samples were then vortexed, the aqueous layer separated and supplemented with 50μl 5M NaCl and a second phenol chloroform extraction performed on the extracted aqueous layer. DNA was precipitated with the addition of one volume of 100% isopropanol and one-tenth volume of 3M sodium acetate and incubating at -20°C overnight. The DNA pellet was washed with 70% ethanol, dried and resuspended in nuclease-free water. Mtb genomes were then quantified using Taqman Universal Master Mix II (Life Technologies) and previously published *sigF* primer-probe combination [55]. Each sample was amplified in triplicate using an ABI Systems 7900HT machine. Chromosomal equivalents (CEQ) were quantified by comparing the samples with a standard curve derived from serial dilution of Mtb genomes prepared from liquid culture. Our detection limit for the standard curve was 10 copies per reaction. When we calculated the number of genomes for the whole lymph node, our detection limit was 1,000 copies per lymph node.

### Statistical analysis

D’Agostino & Pearson Omnibus normality test was performed on all data described in this manuscript. Since the data were not normally distributed, Nonparametric t-test was used when comparing two groups (Mann-Whitney test). Kruskal-Wallis test was used to compare more than two groups with post hoc analysis Dunn’s multiple test comparisons. P values ≤0.05 were considered significant. Statistical analysis was performed using GraphPad Prism v7 (GraphPad Software, San Diego, CA). For multivariate analysis, JMP Pro v12 (SAS Institute Inc., NC, USA) package was used. Nonparametric Spearman’s rho was calculated for correlations (multivariate analysis) using JMP Pro v12. All CFU data and CEQ data used in CFU/CEQ graphs were transformed by adding 1 to reflect sterile LNs in log-scale graphs.

## Supporting information

S1 Fig Modest positive correlation between LN SUVR and live bacterial burden at necropsy. Each data point is a lymph node. Correlation was determined using Spearman’s rank correlation test.
(TIF)

S2 Fig Comparison of CFU, CEQ and killing (CFU/CEQ) between cynomolgus and rhesus macaques at similar time points post infection. A. Rhesus macaque lymph nodes have fewer live Mtb burden at 11–14 weeks post infection compared to cynomolgus macaques. B. Overall, there is little difference in the total (live+dead) Mtb burden in rhesus and cynomolgus macaque lymph nodes at the various time points post infection analyzed. C. Cynomolgus macaque lymph nodes are better at killing Mtb than rhesus macaque lymph nodes at 11–14 weeks post infection. Each data point is a lymph node. Each color is a macaque. Open symbols represent sterile lymph nodes. Statistics are Mann-Whitney.
(TIF)

S3 Fig LN CFU (A) and CFU/CEQ (B) of cynomolgus macaques with active disease, controlling disease and rhesus macaques at 16–29 weeks post-infection. Statistical test is Kruskal Wallis with Dunn’s multiple comparisons test.
(TIF)

S4 Fig Isoniazid treatment for 2 months does not significantly change CEQ in lymph nodes. A. CEQ is similar between INH-treated (N = 4) and control (N = 7) macaques. B. CEQ is similar between sterile and nonsterile lymph nodes with granulomas in INH-treated macaques. C. Greater killing capacity of sterile lymph nodes compared to nonsterile lymph nodes in INH-treated macaques. Each data point is a lymph node. Statistics are Mann-Whitney for A; there were insufficient samples for statistics in B and C.
(TIF)

S5 Fig Proportion of thoracic lymph nodes that had granuloma by gross and microscopic examination at necropsy. Time points shown are necropsy time points for cynomolgus and rhesus macaques.
(TIF)

S6 Fig Comparison of immune responses in peripheral and thoracic LNs. Peripheral (n = 14) and thoracic LNs (n = 27) from 7 animals were stimulated with ESAT6 and CFP10 peptides. A. Frequency of CD3+ T cells. B. Frequency of CD4+ T cells. C. Proliferative capacity of T cells measured by Ki67 in CD3+ and CD8+ T cells. Ki67+ T cells are significantly higher in thoracic LNs than in peripheral LNs. Each symbol is a LN. Peripheral LNs are in green and thoracic LNs are in blue. Statistics are Mann-Whitney.
(TIF)

S7 Fig Correlation between extrapulmonary score (extent of extrapulmonary disease at necropsy) and LN necropsy score and bacterial burden. A. There is a moderate positive correlation between extrapulmonary score and lymph node necropsy score [35] in rhesus macaques but not in cynomolgus macaques. B. No relationship between extrapulmonary score and total LN CFU in cynomolgus and rhesus macaques. Each data point is a macaque. Statistical test is F test.
(TIF)

S8 Fig Reduction of bacterial burden in lymph nodes (A) and lung granulomas (B) of cynomolgus macaques after 2 months of linezolid (LZD) therapy (data from study [57]). In box under each graph is the median for each group, used to calculate fold reduction in text. Control n = 8, LZD n = 5. Each data point is a granuloma or a lymph node. Statistical test is Mann-Whitney.
(TIF)

S1 Table List of macaques used in this study (CFU, CEQ, CFU/CEQ). (DOCX)

S2 Table List of macaques used for immunological assays. (DOCX)

S3 Table Cytokine responses in CFU+ and CFU- thoracic LNs. Panel A shows T cell, B cell and macrophage cytokines in CFU+ and CFU- LNs in response to Mtb-specific antigens, ESAT-6 and CFP-10. Panel B shows T cell, B cell and macrophage cytokines in CFU+ and CFU- LNs in response to non-specific stimulation, PDBu and ionomycin. Panel C shows the correlation between bacterial burden per LN and T cell, B cell and macrophage cytokine responses to Mtb-specific antigens, ESAT-6 and CFP-10.
(DOCX)

S4 Table T cell cytokines and proliferative capacity in response to PDBu and ionomycin in thoracic and peripheral LNs. (DOCX)

S5 Table T cell cytokines in response to Mtb-specific antigens (ESAT-6 and CFP-10) in differentially effaced LNs. MCT = multiple comparison test.
(DOCX)
