# Supplementary figures and images for: Lymph nodes are sites of prolonged bacterial persistence during Mycobacterium tuberculosis infection in macaques
Source: PLoS Pathog. 2018 Nov 1;14(11):e1007337. doi: 10.1371/journal.ppat.1007337 (PMC6211753; doi:10.1371/journal.ppat.1007337)

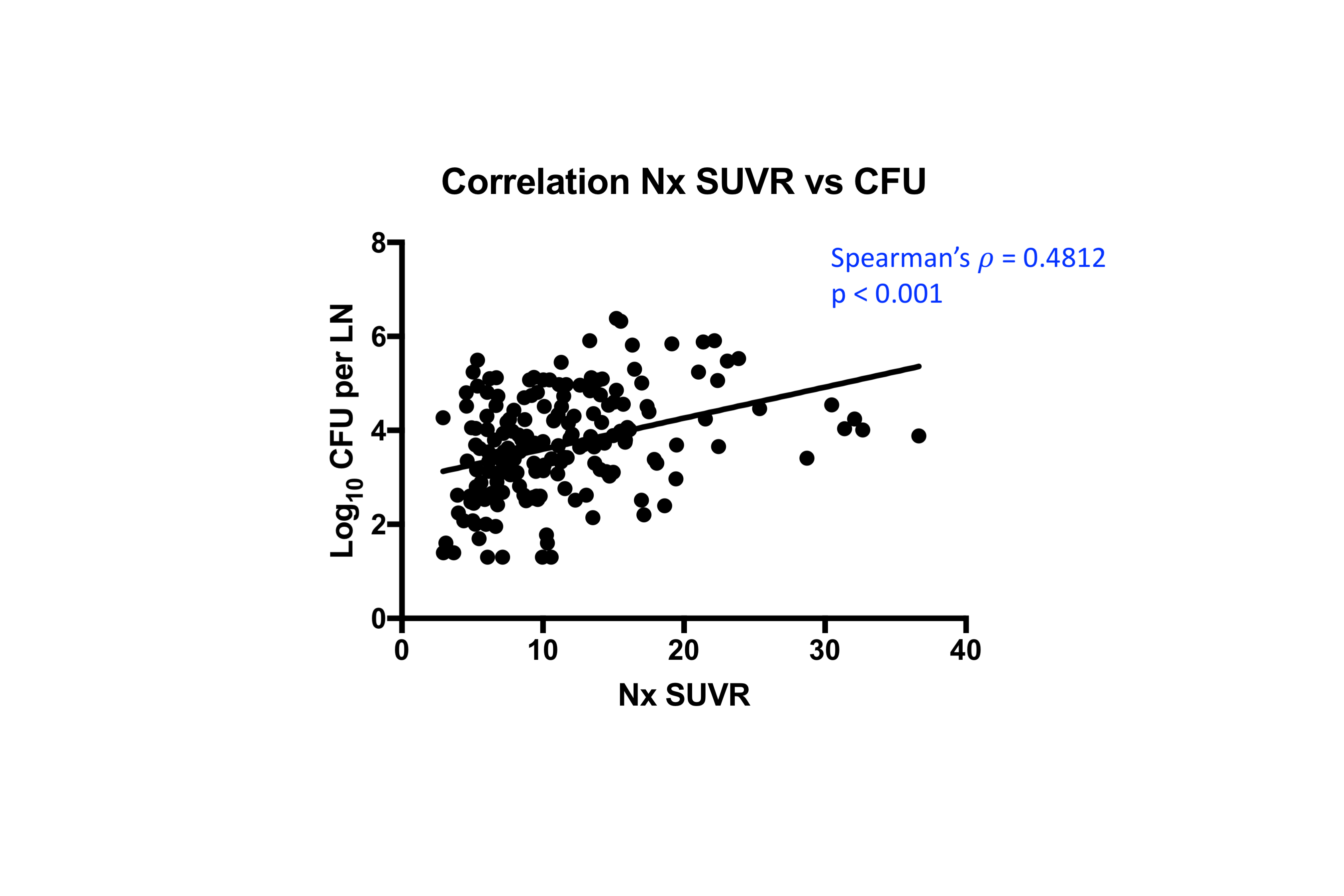

Supplement: S1 Fig — Each data point is a lymph node. Correlation was determined using Spearman’s rank correlation test. (TIF) [file ppat.1007337.s001.tif]

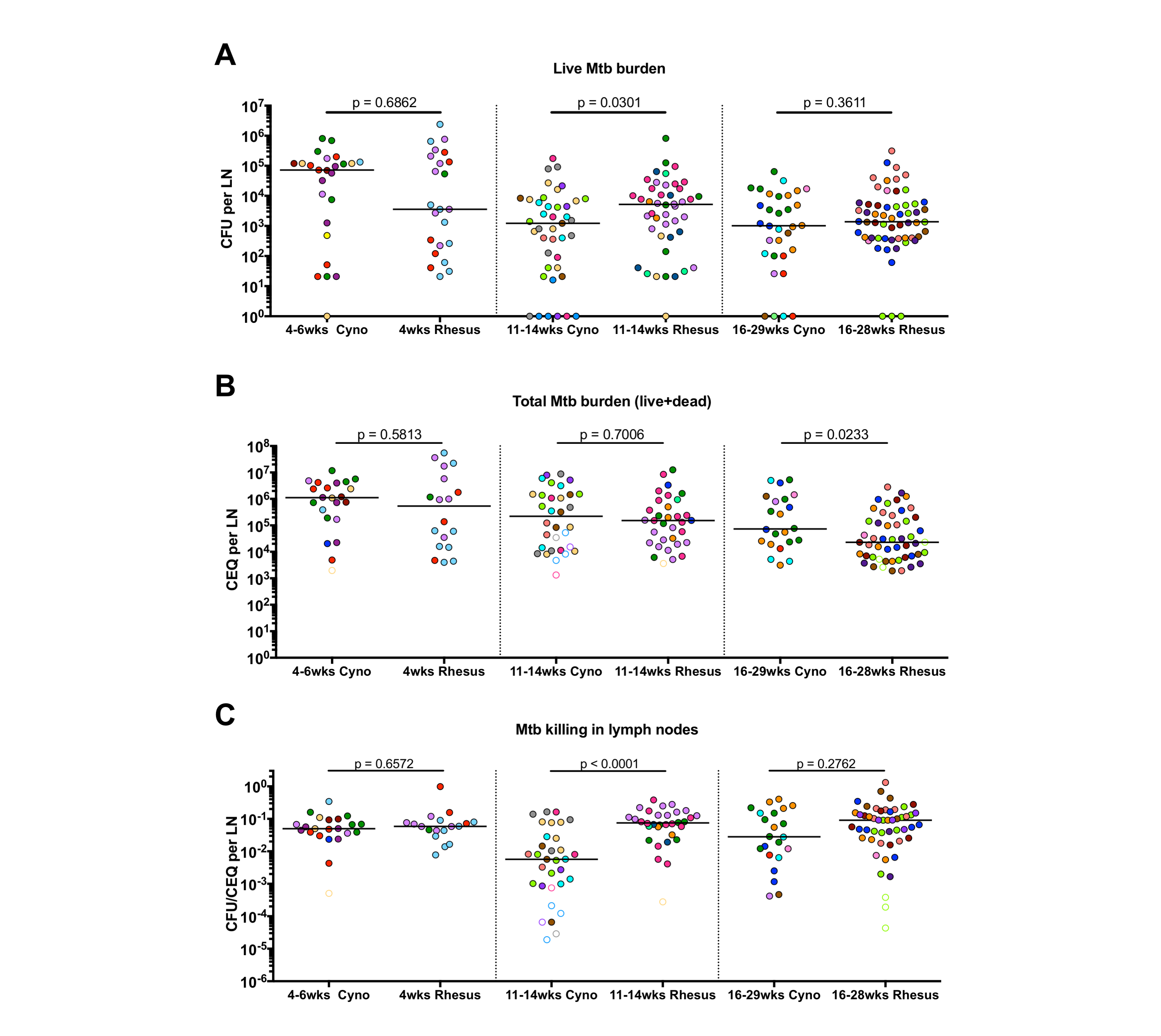

Supplement: S2 Fig — A. Rhesus macaque lymph nodes have fewer live Mtb burden at 11–14 weeks post infection compared to cynomolgus macaques. B. Overall, there is little difference in the total (live+dead) Mtb burden in rhesus and cynomolgus macaque lymph nodes at the various time points post infection analyzed. C. Cynomolgus macaque lymph nodes are better at killing Mtb than rhesus macaque lymph nodes at 11–14 weeks post infection. Each data point is a lymph node. Each color is a macaque. Open symbols represent sterile lymph nodes. Statistics are Mann-Whitney. (TIF) [file ppat.1007337.s002.tif]

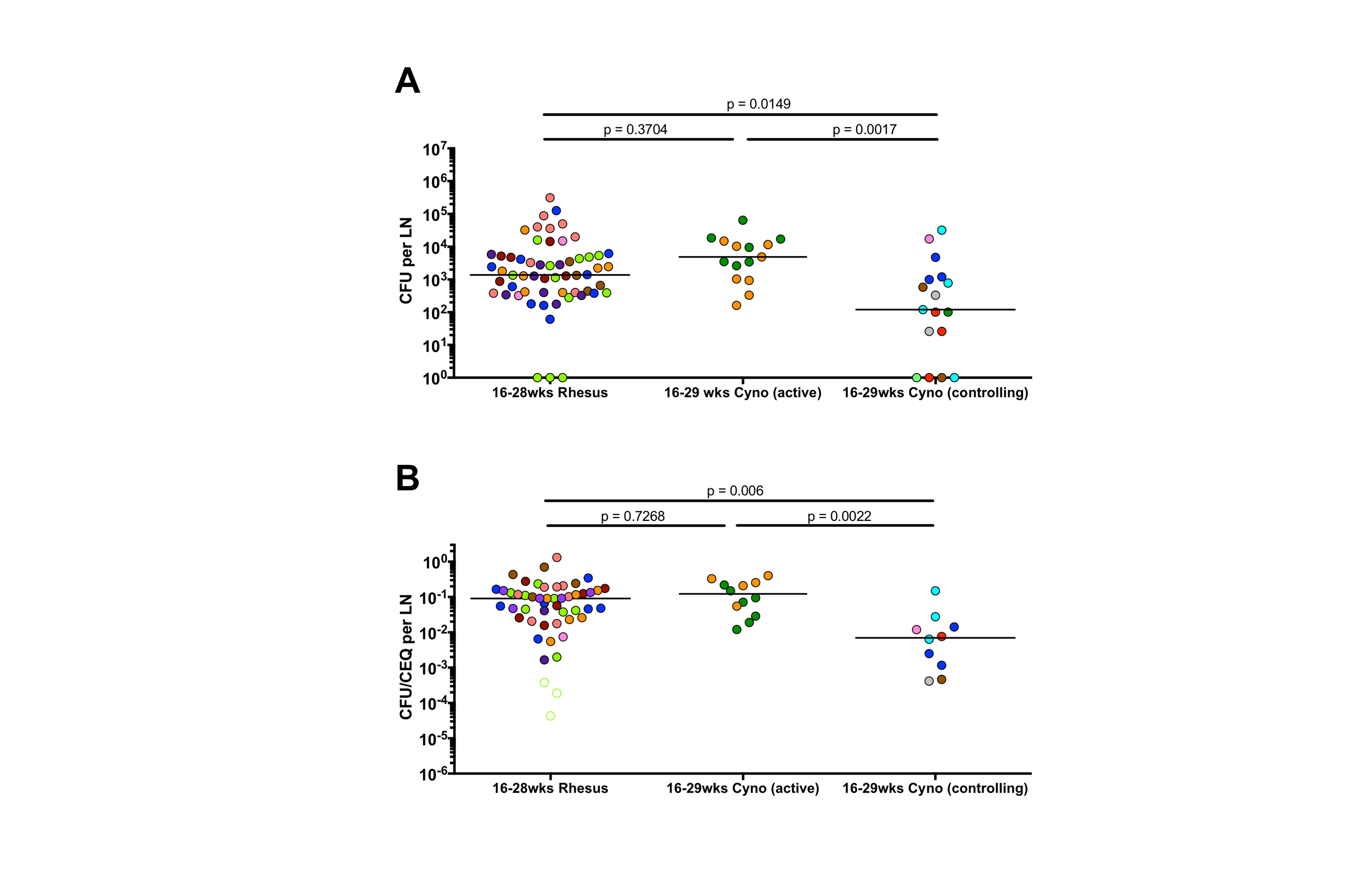

Supplement: S3 Fig — Statistical test is Kruskal Wallis with Dunn’s multiple comparisons test. (TIF) [file ppat.1007337.s003.tif]

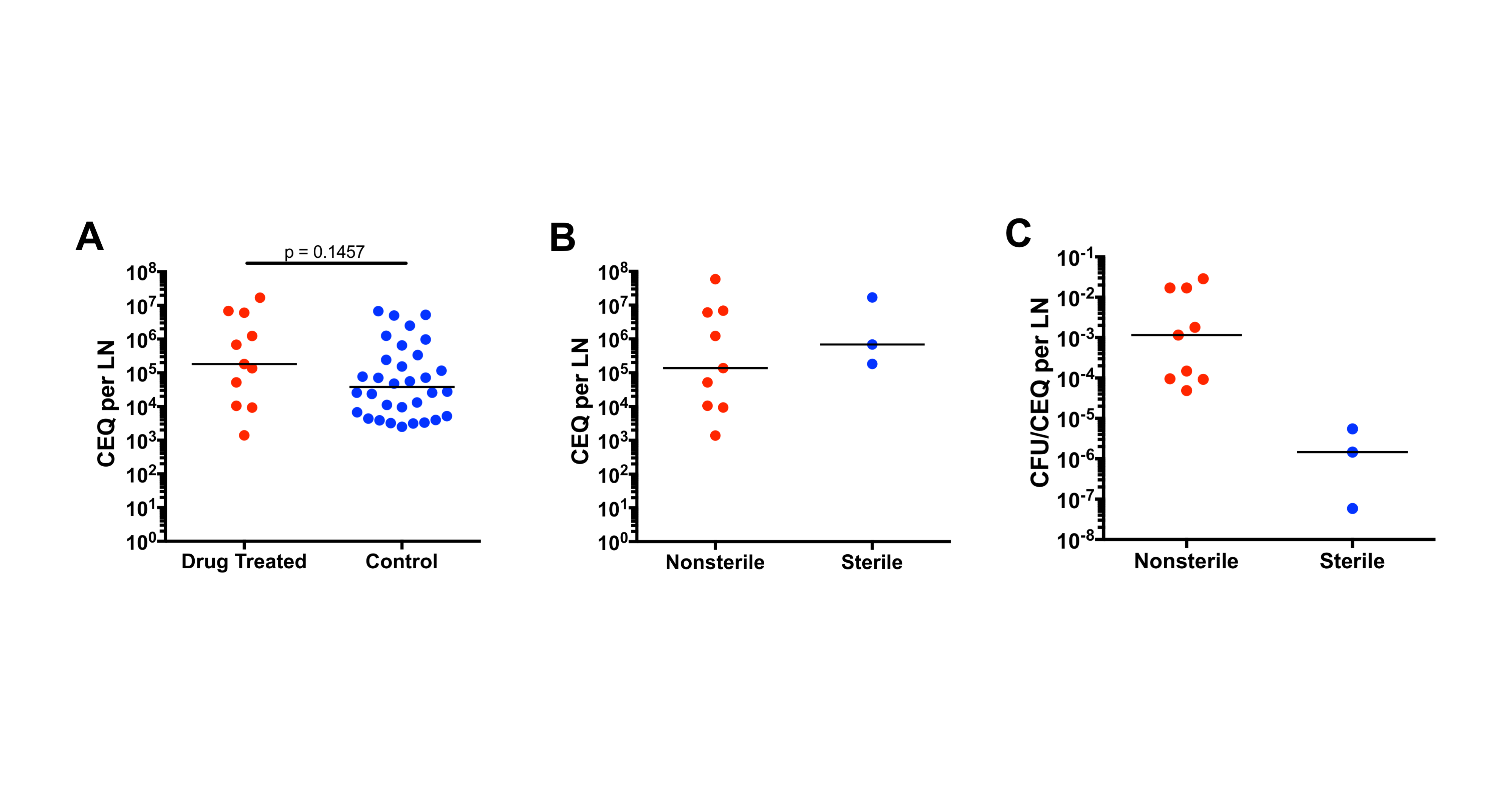

Supplement: S4 Fig — A. CEQ is similar between INH-treated (N = 4) and control (N = 7) macaques. B. CEQ is similar between sterile and nonsterile lymph nodes with granulomas in INH-treated macaques. C. Greater killing capacity of sterile lymph nodes compared to nonsterile lymph nodes in INH-treated macaques. Each data point is a lymph node. Statistics are Mann-Whitney for A; there were insufficient samples for statistics in B and C. (TIF) [file ppat.1007337.s004.tif]

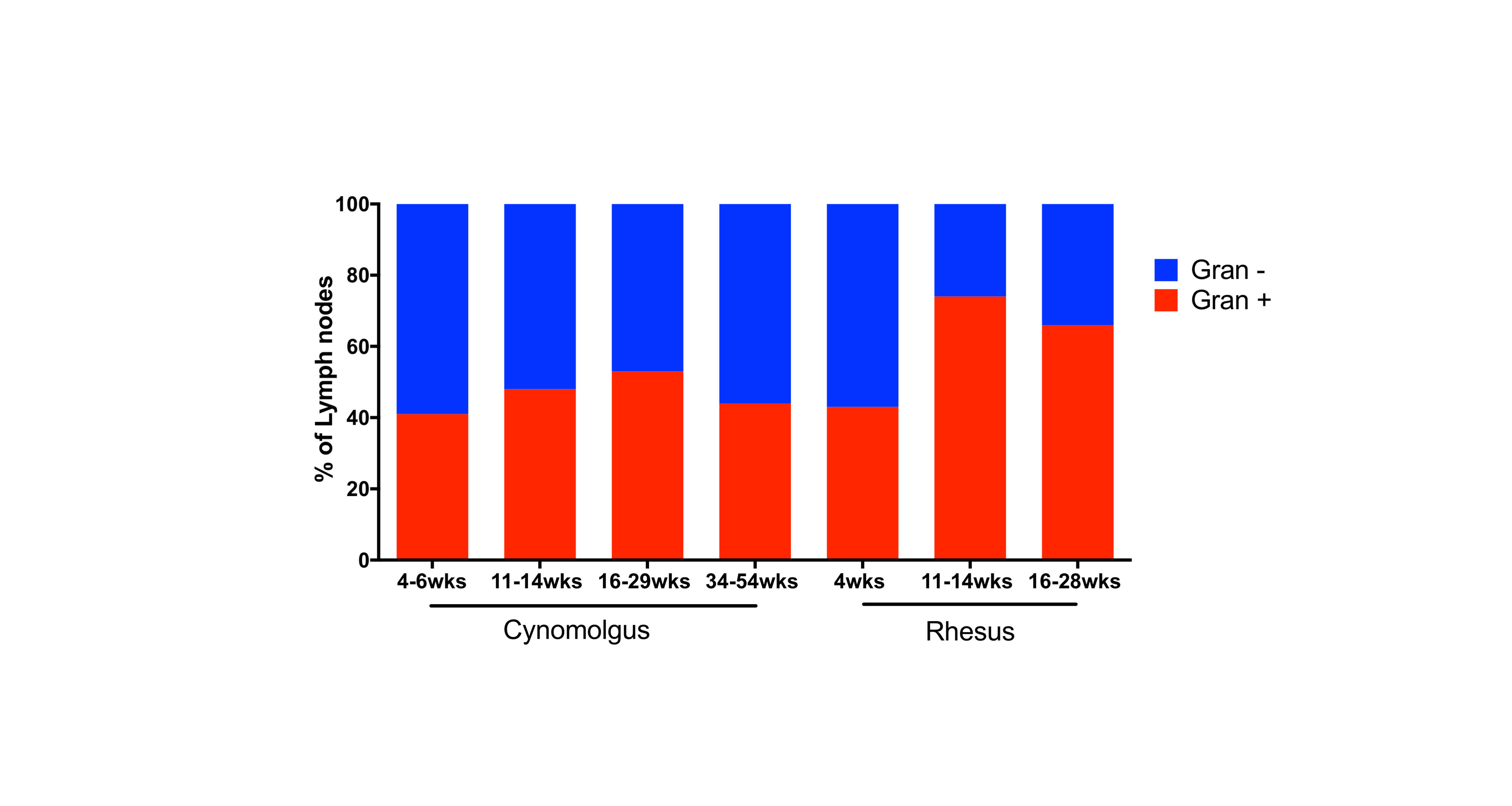

Supplement: S5 Fig — Time points shown are necropsy time points for cynomolgus and rhesus macaques. (TIF) [file ppat.1007337.s005.tif]

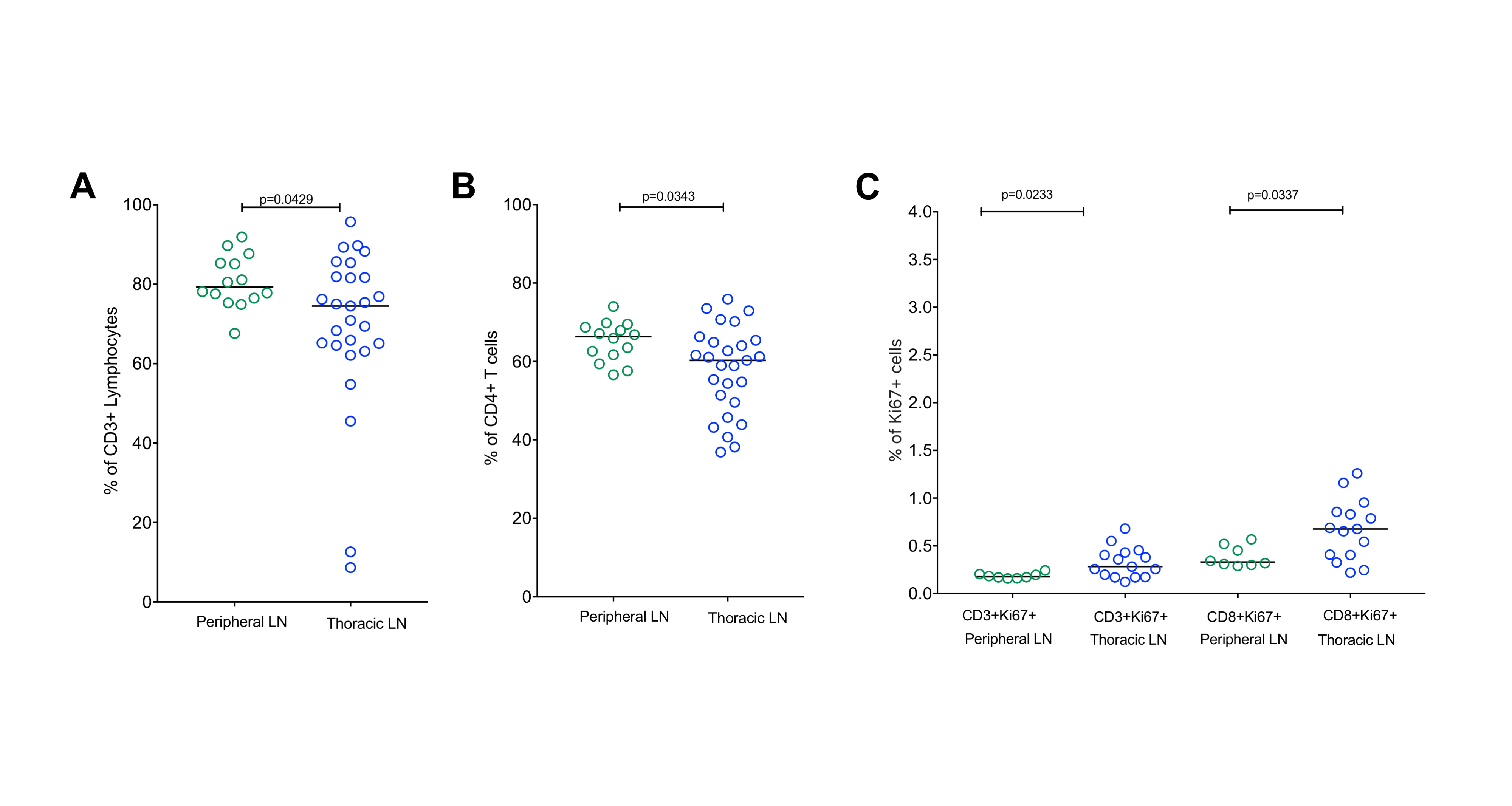

Supplement: S6 Fig — Peripheral (n = 14) and thoracic LNs (n = 27) from 7 animals were stimulated with ESAT6 and CFP10 peptides. A. Frequency of CD3+ T cells. B. Frequency of CD4+ T cells. C. Proliferative capacity of T cells measured by Ki67 in CD3+ and CD8+ T cells. Ki67+ T cells are significantly higher in thoracic LNs than in peripheral LNs. Each symbol is a LN. Peripheral LNs are in green and thoracic LNs are in blue. Statistics are Mann-Whitney. (TIF) [file ppat.1007337.s006.tif]

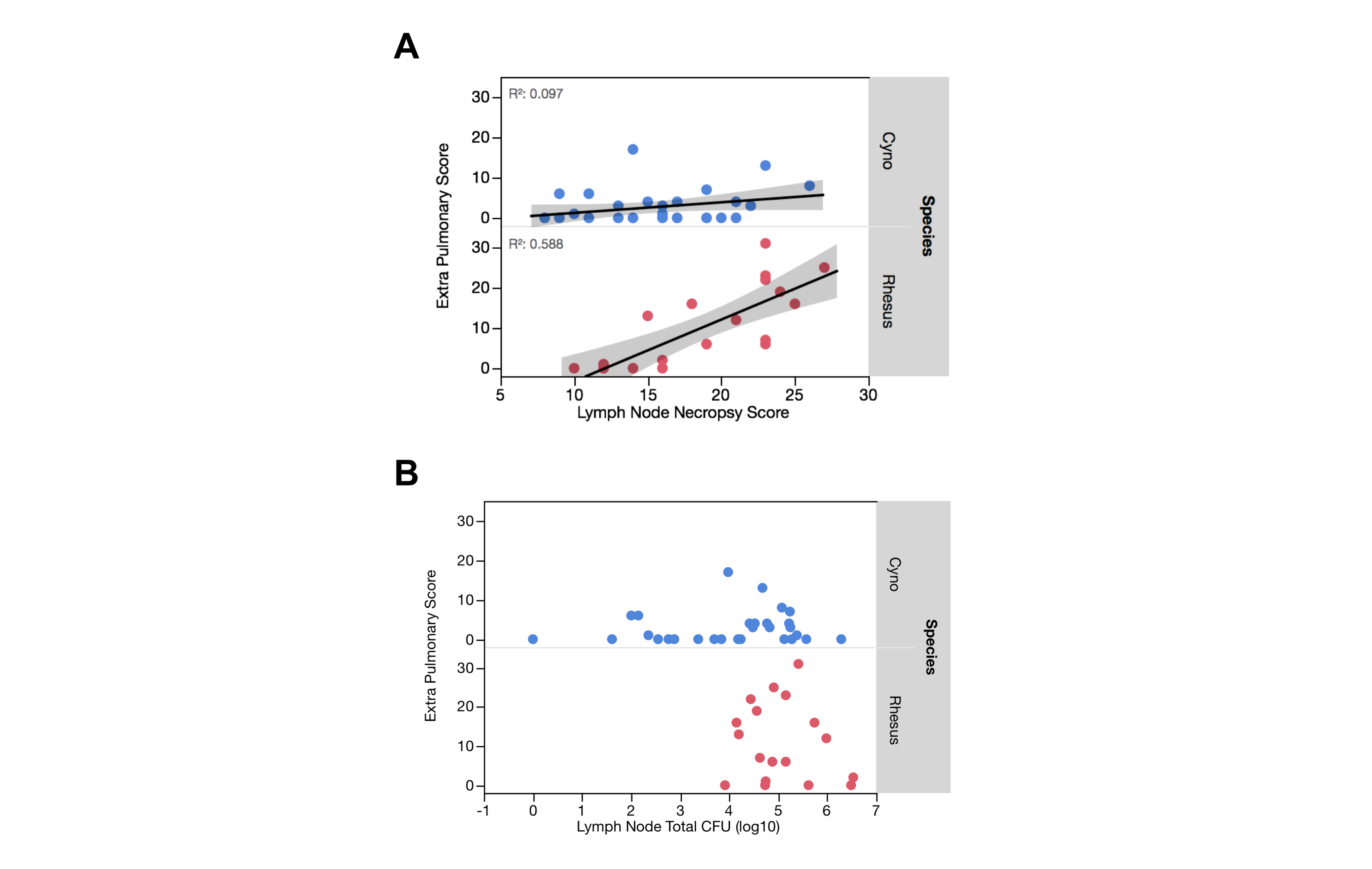

Supplement: S7 Fig — A. There is a moderate positive correlation between extrapulmonary score and lymph node necropsy score [35] in rhesus macaques but not in cynomolgus macaques. B. No relationship between extrapulmonary score and total LN CFU in cynomolgus and rhesus macaques. Each data point is a macaque. Statistical test is F test. (TIF) [file ppat.1007337.s007.tif]

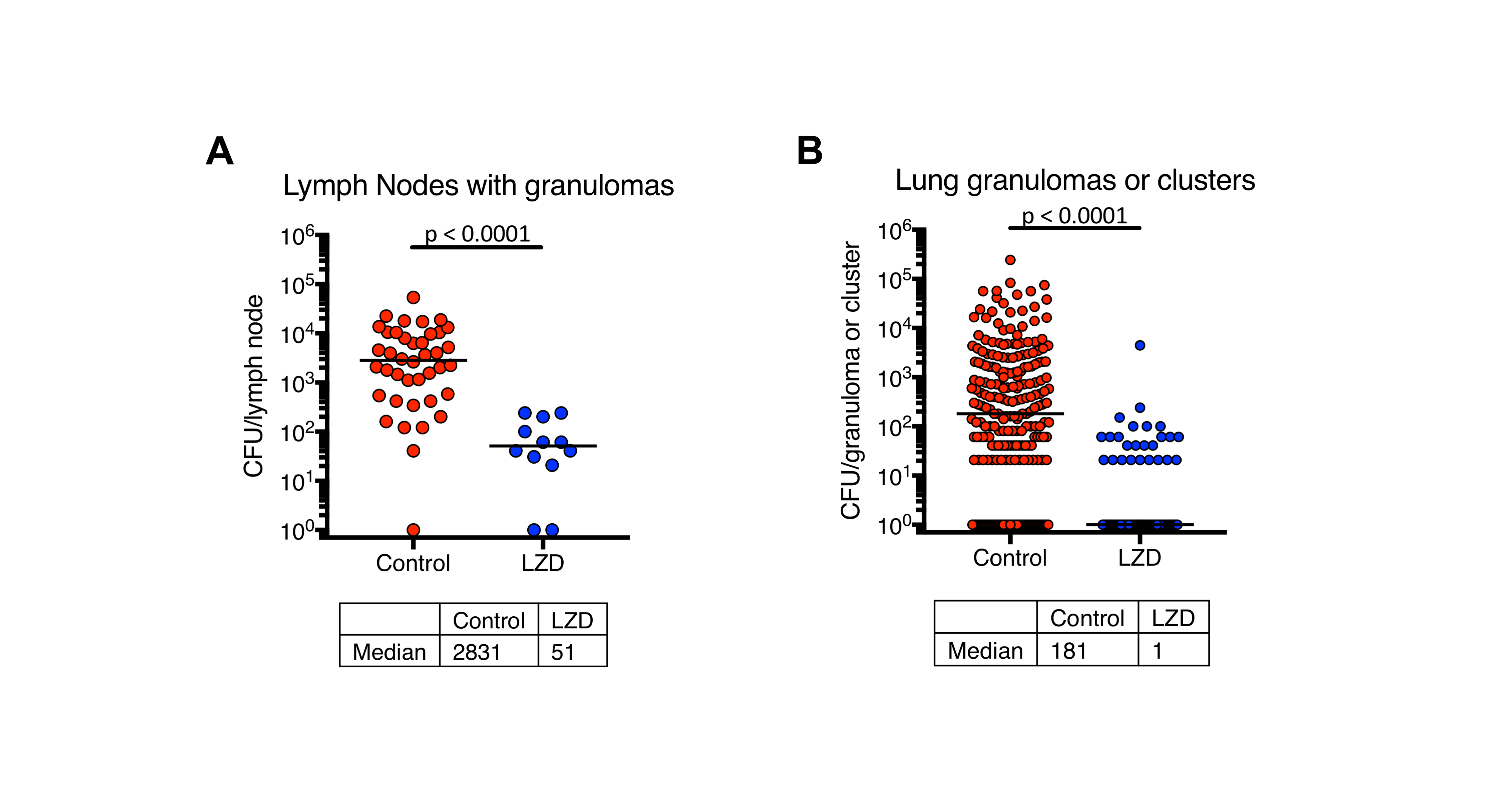

Supplement: S8 Fig — In box under each graph is the median for each group, used to calculate fold reduction in text. Control n = 8, LZD n = 5. Each data point is a granuloma or a lymph node. Statistical test is Mann-Whitney. (TIF) [file ppat.1007337.s008.tif]
