# Supplementary material for: Lymph nodes are sites of prolonged bacterial persistence during Mycobacterium tuberculosis infection in macaques
Source: PLoS Pathog. 2018 Nov 1;14(11):e1007337. doi: 10.1371/journal.ppat.1007337 (PMC6211753; doi:10.1371/journal.ppat.1007337)
Supplement: S1 Table — (DOCX) [file ppat.1007337.s009.docx]

| **Monkey ID** | **Species** | **Days post-infection** | **Wks post-infection** | **Date infected** | **Dose** | **Necropsy date** | **PET CT scans (wks pi)** | **Reference** |
| --- | --- | --- | --- | --- | --- | --- | --- | --- |
| **1414** | Cyno | 26 | 4 | 18-Jun-14 | 4.7 | 14-Jul-14 | 3 | unpublished |
| **1014** | Cyno | 28 | 4 | 23-May-14 | 1 | 20-Jun-14 | 2, 4 | unpublished |
| **1514** | Cyno | 28 | 4 | 18-Jun-14 | 4.7 | 16-Jul-14 | 4 | unpublished |
| **19015** | Cyno | 29 | 4 | 17-Nov-15 | 6 | 16-Dec-15 | 2, 4 | [65] |
| **1214** | Cyno | 31 | 4 | 23-May-14 | 1 | 23-Jun-14 | 2, 4 | unpublished |
| **8715** | Cyno | 36 | 5 | 12-Jan-16 | 2 | 17-Feb-16 | 2, 3, 4 | unpublished |
| **8915** | Cyno | 36 | 5 | 12-Jan-16 | 2 | 17-Feb-16 | 2, 3, 5 | unpublished |
| **8815** | Cyno | 41 | 6 | 12-Jan-16 | 2 | 22-Feb-16 | 2, 3, 4, 5 | unpublished |
| **2116**** | Cyno | 74 | 11 | 25-Feb-16 | 3 | 9-May-16 | 4, 6, 8, 10 | [35] |
| **9814** | Cyno | 78 | 11 | 26-Aug-14 | 6 | 12-Nov-14 | 3, 6, 11 | [35] |
| **1114** | Cyno | 81 | 12 | 28-Aug-14 | 2.7 | 17-Nov-14 | 2, 4, 6, 8, 10, 11 | unpublished |
| **1314** | Cyno | 81 | 12 | 28-Aug-14 | 2.7 | 17-Nov-14 | 2, 4, 6, 8, 10, 11 | unpublished |
| **17413** | Cyno | 83 | 12 | 28-Aug-14 | 2.7 | 19-Nov-14 | 2, 3, 4, 6, 8, 10, 12 | unpublished |
| **616** | Cyno | 88 | 13 | 18-Feb-16 | 11 | 16-May-16 | 4, 6, 8, 12 | [35] |
| **13716** | Cyno | 88 | 13 | 14-Jul-16 | 5 | 10-Oct-16 | 4, 6, 8, 12 | [35] |
| **1714** | Cyno | 91 | 13 | 18-Jun-14 | 4.7 | 17-Sep-14 | 4, 6, 8, 10, 13 | unpublished |
| **916** | Cyno | 95 | 14 | 18-Feb-16 | 11 | 23-May-16 | 4, 5, 8, 13 | [35] |
| **2016** | Cyno | 109 | 16 | 25-Feb-16 | 3 | 13-Jun-16 | 4, 6, 8, 15 | [35] |
| **21112** | Cyno | 133 | 19 | 7-Oct-13 | 12 | 17-Feb-14 | 2, 4, 5, 6, 9, 13, 17, 19 | [35] |
| **20512** | Cyno | 143 | 20 | 12-Jul-13 | 6 | 2-Dec-13 | 2, 3, 4, 8, 12, 16, 20 | [35] |
| **20712** | Cyno | 152 | 22 | 12-Jul-13 | 6 | 11-Dec-13 | 1, 3, 4, 8, 12, 16, 21 | [35] |
| **16213** | Cyno | 163 | 23 | 14-Apr-14 | 15.2 | 24-Sep-14 | 3, 4, 5, 7, 10, 15, 23 | [35] |
| **16013** | Cyno | 170 | 24 | 14-Apr-14 | 15.2 | 1-Oct-14 | 2, 3, 4, 6, 10, 15, 24 | [35] |
| **16113** | Cyno | 170 | 24 | 14-Apr-14 | 15.2 | 1-Oct-14 | 2, 3, 4, 6, 10, 15, 24 | [35] |
| **20715** | Cyno | 174 | 25 | 22-Dec-15 | 12.5 | 13-Jun-16 | 4, 9, 12, 20, 24 | unpublished |
| **20915** | Cyno | 202 | 29 | 22-Dec-14 | 12.5 | 11-Jul-16 | 4, 8, 12, 17, 20, 24, 28 | unpublished |
| **9811** | Cyno | 238 | 34 | 17-Oct-11 | 20 | 11-Jun-12 | 3, 6, 8, 12, 16, 20, 24, 28, 34 | [34, 35, 65] |
| **15712** | Cyno | 293 | 42 | 11-Dec-12 | 12 | 30-Sep-13 | 40 | unpublished |
| **17211** | Cyno | 328 | 47 | 31-Jul-12 | 6 | 24-Jun-13 | 46 | [34, 58, 65] |
| **2312** | Cyno | 330 | 47 | 31-Jul-12 | 6 | 26-Jun-13 | 47 | [34, 58, 65] |
| **15812** | Cyno | 336 | 48 | 11-Mar-13 | 28 | 10-Feb-14 | 31, 40, 46, 48 | unpublished |
| **2612** | Cyno | 379 | 54 | 21-Aug-12 | 8 | 4-Sep-13 | 54 | [34, 56, 65] |
| **5614** | Rhesus | 26 | 4 | 18-Jun-14 | 4.7 | 14-Jul-14 | 3 | unpublished |
| **5714** | Rhesus | 28 | 4 | 18-Jun-14 | 4.7 | 16-Jul-14 | 4 | unpublished |
| **17115** | Rhesus | 28 | 4 | 19-Oct-15 | 5.3 | 16-Nov-15 | 3, 4 | unpublished |
| **17215** | Rhesus | 30 | 4 | 19-Oct-15 | 5.3 | 18-Nov-15 | 3, 4 | unpublished |
| **915**** | Rhesus | 78 | 11 | 7-Apr-15 | 8 | 24-Jun-15 | 3, 8, 11 | [35] |
| **6114** | Rhesus | 80 | 11 | 23-May-14 | 1 | 11-Aug-14 | 2, 4, 8, 10, 11 | unpublished |
| **16915** | Rhesus | 86 | 12 | 12-Oct-15 | 7 | 6-Jan-16 | 3, 7, 12 | [35] |
| **5414** | Rhesus | 89 | 13 | 18-Jun-14 | 4.7 | 15-Sep-14 | 4, 6, 8, 10, 12 | unpublished |
| **5814** | Rhesus | 91 | 13 | 18-Jun-14 | 4.7 | 17-Sep-14 | 4, 6, 8, 10, 13 | unpublished |
| **1415**** | Rhesus | 98 | 14 | 16-Apr-15 | 15 | 23-Jul-15 | 4, 8, 9, 11, 14 | [35] |
| **7614**** | Rhesus | 99 | 14 | 27-May-14 | 8 | 3-Sep-14 | 2, 4, 6, 8, 11, 14 | [35] |
| **613** | Rhesus | 110 | 16 | 13-Mar-13 | 18 | 1-Jul-13 | 2, 4, 6, 8, 12, 15 | [35] |
| **813** | Rhesus | 112 | 16 | 13-Mar-13 | 18 | 3-Jul-13 | 2, 4, 6, 8, 12, 16 | [35] |
| **713** | Rhesus | 117 | 17 | 13-Mar-13 | 18 | 8-Jul-13 | 2, 4, 7, 8, 12, 16 | [35] |
| **2315** | Rhesus | 151 | 22 | 16-Apr-15 | 15 | 5-Aug-15 | 4, 8, 13, 16, 19, 21 | [35] |
| **1215** | Rhesus | 154 | 22 | 7-Apr-15 | 8 | 8-Sep-15 | 4, 8, 12, 16, 20, 21 | [35] |
| **6914** | Rhesus | 181 | 26 | 27-May-14 | 8 | 24-Nov-14 | 2, 4, 7, 8, 12, 17, 20, 25 (CT only) | [35] |
| **8214** | Rhesus | 186 | 27 | 29-May-14 | 10 | 1-Dec-14 | 4, 6, 8, 12, 16, 20, 26 | [35] |
| **7114** | Rhesus | 195 | 28 | 27-May-14 | 8 | 8-Dec-14 | 2, 4, 7, 8, 12, 16, 20, 27 | [35] |

** Euthanized before study endpoint
