# Supplementary material for: Lymph nodes are sites of prolonged bacterial persistence during Mycobacterium tuberculosis infection in macaques
Source: PLoS Pathog. 2018 Nov 1;14(11):e1007337. doi: 10.1371/journal.ppat.1007337 (PMC6211753; doi:10.1371/journal.ppat.1007337)
Supplement: S2 Table — (DOCX) [file ppat.1007337.s010.docx]

| **Monkey ID** | **Species** | **Days post-infection** | **Wks post-infection** | **Date infected** | **Dose** | **Nx date** | **Reference** |
| --- | --- | --- | --- | --- | --- | --- | --- |
| **2312** | Cyno | 330 | 47 | 31-Jul-12 | 6 | 26-Jun-13 | [34, 65] |
| **2412** | Cyno | 83 | 12 | 8-May-12 | 4 | 30-Jul-12 | [34, 65] |
| **2512** | Cyno | 85 | 12 | 5-Jun-12 | 8 | 29-Aug-12 | [34, 65] |
| **2612** | Cyno | 379 | 54 | 21-Aug-12 | 8 | 4-Sep-13 | [34, 65] |
| **2712** | Cyno | 384 | 55 | 21-Aug-12 | 8 | 9-Sep-13 | [34, 65] |
| **5716** | Cyno | 30 | 4 | 29-Mar-16 | 10 | 28-Apr-16 | Unpublished |
| **6610** | Cyno | 601 | 86 | 24-Mar-11 | 45 | 14-Nov-12 | [65] |
| **9511** | Cyno | 198 | 28 | 24-Oct-11 | 12 | 9-May-12 | [34, 65] |
| **9711** | Cyno | 175 | 25 | 17-Oct-11 | 20 | 9-Apr-12 | [34, 65] |
| **15312** | Cyno | 124 | 18 | 8-Mar-13 | 40 | 10-Jul-13 | [34] |
| **15712** | Cyno | 293 | 42 | 11-Dec-12 | 12 | 30-Sep-13 | [65] |
| **16113** | Cyno | 170 | 24 | 14-Apr-14 | 15.2 | 1-Oct-14 | [34] |
| **16213** | Cyno | 163 | 23 | 14-Apr-14 | 15.2 | 24-Sep-14 | [34] |
| **17111** | Cyno | 84 | 12 | 1-May-12 | 8 | 24-Jul-12 | [65] |
| **17211** | Cyno | 328 | 47 | 31-Jul-12 | 6 | 24-Jun-13 | [65] |
| **19915** | Cyno | 28 | 4 | 14-Mar-16 | 5 | 11-Apr-16 | Unpublished |
| **20212** | Cyno | 72 | 10 | 18-Feb-13 | 4 | 1-May-13 | [65] |
| **20612** | Cyno | 72 | 10 | 25-Feb-13 | 6 | 8-May-13 | [65] |
| **20712** | Cyno | 152 | 22 | 12-Jul-13 | 6 | 11-Dec-13 | [65] |
| **20912** | Cyno | 72 | 10 | 4-Mar-13 | 4 | 15-May-13 | [65] |
| **22410** | Cyno | 580 | 83 | 26-Apr-11 | 46 | 26-Nov-12 | [65] |
| **22510** | Cyno | 297 | 42 | 24-May-11 | 42 | 16-Mar-12 | [65] |
| **22610** | Cyno | 538 | 77 | 26-Apr-11 | 46 | 15-Oct-12 | [65] |
| **22810** | Cyno | 386 | 55 | 17-May-11 | 22 | 6-Jun-12 | [65] |
