# Supplementary material for: Lymph nodes are sites of prolonged bacterial persistence during Mycobacterium tuberculosis infection in macaques
Source: PLoS Pathog. 2018 Nov 1;14(11):e1007337. doi: 10.1371/journal.ppat.1007337 (PMC6211753; doi:10.1371/journal.ppat.1007337)
Supplement: S3 Table — Panel A shows T cell, B cell and macrophage cytokines in CFU+ and CFU- LNs in response to Mtb-specific antigens, ESAT-6 and CFP-10. Panel B shows T cell, B cell and macrophage cytokines in CFU+ and CFU- LNs in response to non-specific stimulation, PDBu and ionomycin. Panel C shows the correlation between bacterial burden per LN and T cell, B cell and macrophage cytokine responses to Mtb-specific antigens, ESAT-6 and CFP-10. (DOCX) [file ppat.1007337.s011.docx]

| Cell Type/Cytokine | Panel A | | | Panel B | | | Panel C | | |
| --- | --- | --- | --- | --- | --- | --- | --- | --- | --- |
|  | Thoracic LNs with granuloma: CFU+ vs CFU- (ESAT6+CFP10) | | | Thoracic LNs with granuloma: CFU+ vs CFU- (PDBu and ionomycin) | | | Thoracic LN correlation between CFU burden/LN and variable* (ESAT6+CFP10) | | |
|  | CFU+ (Median, n of LN) | CFU- (Median, n of LN) | p value | CFU+ (Median, n of LN) | CFU- (Median, n of LN) | p value | Variable* | Spearman ρ | Prob>\|ρ\| |
| CD3+ | 74.5, n=28 | 77.95, n=20 | 0.1518 | 71.55, n=22 | 75.8, n=14 | 0.1942 | CD3+ | -0.2798 | 0.0541 |
| CD3+ IFNg | 0.288, n=28 | 0.525, n=20 | 0.3111 | **3.28, n=22** | **7.825, n=14** | **0.0227** | CD3+ IFNg | -0.1637 | 0.2662 |
| CD3+ IL-2 | 0.3175, n=22 | 0.339, n=20 | 0.6627 | 2.91, n=17 | 7, n=14 | 0.1579 | CD3+ IL-2 | -0.1437 | 0.3639 |
| CD3+ TNF | 0.892, n=28 | 0.551, n=20 | 0.078 | 3.665, n=22 | 9.26, n=14 | 0.0663 | CD3+ TNF | 0.2037 | 0.1649 |
| CD3+ IL-17 | 0.549, n=27 | 0.577, n=18 | 0.4802 | 2.04, n=22 | 3.25, n=13 | 0.3185 | CD3+ IL-17 | -0.1555 | 0.3078 |
| CD3+ IL-10 | 0.958, n=13 | 1.685, n=14 | 0.7564 | 0.725, n=14 | 2.8, n=11 | 0.4342 | CD3+ IL-10 | 0.0201 | 0.9208 |
| CD3+ Ki67 | 1.285, n=6 | 0.614, n=11 | 0.7864 | 0.2785, n=8 | 1.69, n=9 | 0.2766 | CD3+ Ki67 | 0.0201 | 0.939 |
| CD4+ | 61, n=28 | 55.3, n=20 | 0.4872 | 59.75, n=22 | 69.05, n=14 | 0.178 | CD4+ | 0.0915 | 0.5361 |
| CD4+ IFNg | 0.3165, n=28 | 0.4245, n=20 | 0.7522 | 2.425, n=22 | 4.01, n=14 | 0.0604 | CD4+ IFNg | -0.069 | 0.6414 |
| CD4+ IL-2 | 0.1495, n=22 | 0.2215, n=20 | 0.3391 | 3.62, n=17 | 8.15, n=14 | 0.1179 | CD4+ IL-2 | -0.2455 | 0.1171 |
| CD4+ TNF | **0.955, n=28** | **0.4025, n=20** | **0.019** | 3.53, n=22 | 10.85, n=14 | 0.0825 | CD4+ TNF | **0.2892** | **0.0462** |
| CD4+ IL-17 | 0.415, n=27 | 0.37, n=18 | 0.959 | 1.915, n=22 | 3.36, n=13 | 0.1055 | CD4+ IL-17 | -0.0493 | 0.7479 |
| CD4+ IL-10 | 0.882, n=13 | 0.2755, n=14 | 0.1408 | 0.3075, n=14 | 2.23, n=11 | 0.2671 | CD4+ IL-10 | 0.2879 | 0.1453 |
| CD4+ Ki67 | 1.24, n=6 | 0.413, n=11 | 0.5908 | 0.2085, n=8 | 1.33, n=9 | 0.1996 | CD4+ Ki67 | 0.1004 | 0.7015 |
| CD8+ | 26.8, n=28 | 25.4, n=20 | 0.9463 | 23.05, n=22 | 20.8, n=14 | 0.6137 | CD8+ | 0.0312 | 0.8333 |
| CD8+ IFNg | 0.225, n=28 | 0.4115, n=20 | 0.5265 | **5.26, n=21** | **16.5, n=14** | **0.0201** | CD8+ IFNg | -0.1113 | 0.4513 |
| CD8+ IL-2 | 0.273, n=22 | 0.44, n=20 | 0.7503 | **1.295, n=16** | **5.125, n=14** | **0.0472** | CD8+ IL-2 | -0.1128 | 0.4768 |
| CD8+ TNF | 0.7145, n=28 | 0.5875, n=20 | 0.3136 | **2.69, n=21** | **10.95, n=14** | **0.026** | CD8+ TNF | 0.0999 | 0.4992 |
| CD8+ IL-17 | 0.394, n=27 | 0.345, n=18 | 0.8051 | 0.845, n=21 | 1.19, n=13 | 0.4052 | CD8+ IL-17 | -0.0315 | 0.8374 |
| CD8+ IL-10 | 1.43, n=13 | 0.6015, n=14 | 0.1373 | 0.348, n=13 | 0.695, n=11 | 0.2284 | CD8+ IL-10 | 0.2151 | 0.2813 |
| CD8+ Ki67 | 1.205, n=6 | 0.905, n=11 | 0.3378 | 0.65, n=8 | 2.15, n=9 | 0.2359 | CD8+ Ki67 | 0.2224 | 0.3909 |
| CD20+ | 15.4, n=19 | 19.6, n=11 | 0.3227 | 17.2, n=17 | 24.3, n=8 | 0.0356 | CD20+ | -0.0365 | 0.8481 |
| CD20+ IFNg | 0.889, n=19 | 0.5, n=11 | 0.545 | 0.483, n=17 | 1.705, n=8 | 0.1527 | CD20+ IFNg | 0.0598 | 0.7534 |
| CD20+ IL-2 | 0.754, n=13 | 1.12, n=11 | 0.833 | **0.3285, n=12** | **2.455, n=8** | **0.0124** | CD20+ IL-2 | -0.0188 | 0.9307 |
| CD20+ TNF | 1.87, n=19 | 0.943, n=11 | 0.1746 | 2.22, n=17 | 4.085, n=8 | 0.0673 | CD20+ TNF | 0.261 | 0.1636 |
| CD20+ IL-17 | 2.41, n=18 | 2.81, n=9 | 0.7144 | 3.86, n=17 | 4.01, n=7 | 0.2598 | CD20+ IL-17 | 0.0716 | 0.7228 |
| CD20+ IL-10 | 0.958, n=13 | 1.685, n=14 | 0.7564 | 0.725, n=14 | 2.8, n=11 | 0.4342 | CD20+ IL-10 | 0.032 | 0.8876 |
| CD11b+ | 14.7, n=8 | 22.1, n=9 | 0.4807 | 17.55, n=10 | 22.1, n=7 | 0.6691 | CD11b+ | -0.1102 | 0.6737 |
| CD11b+ IFNg | 11.2, n=8 | 8.13, n=9 | 0.8148 | **11.4, n=9** | **20, n=7** | **0.0229** | CD11b+ IFNg | -0.0505 | 0.8475 |
| CD11b+ TNF | 12.88, n=8 | 6.44, n=9 | 0.8884 | 9.89, n=9 | 19.5, n=7 | 0.1416 | CD11b+ TNF | -0.2018 | 0.4372 |
| CD11b+ IL-17 | 4.35, n=8 | 14.59, n=4 | 0.1535 | 3.57, n=7 | 15.72, n=4 | 0.2303 | CD11b+ IL-17 | -0.3673 | 0.2402 |
| CD11b+ IL-10 | **3.42, n=8** | **21.8, n=9** | **0.0464** | **0.814, n=9** | **26.5, n=7** | **0.0021** | CD11b+ IL-10 | **-0.5046** | **0.0389** |
