# Supplementary material for: Lymph nodes are sites of prolonged bacterial persistence during Mycobacterium tuberculosis infection in macaques
Source: PLoS Pathog. 2018 Nov 1;14(11):e1007337. doi: 10.1371/journal.ppat.1007337 (PMC6211753; doi:10.1371/journal.ppat.1007337)
Supplement: S4 Table — (DOCX) [file ppat.1007337.s012.docx]

| Cell Type/Cytokine | Peripheral LN (Median, n of LN) | Thoracic LN (Median, n of LN) | p value |
| --- | --- | --- | --- |
| **CD3+** | **79.3, n=14** | **74.5, n=27** | **0.0429** |
| CD3+ IFNg | 0.216, n=14 | 0.249, n=27 | 0.6540 |
| CD3+ IL-2 | 0.712, n=14 | 0.593, n=27 | 0.9293 |
| CD3+ TNF | 0.5135, n=14 | 0.79, n=27 | 0.1586 |
| CD3+ IL-17 | 0.5615, n=14 | 0.363, n=27 | 0.3334 |
| CD3+ IL-10 | 0.258, n=9 | 0.438, n=17 | 0.6340 |
| CD3+ Ki67 | **0.177, n=8** | **0.283, n=18** | **0.0233** |
| CD4+ | **66.35, n=14** | **60.3, n=27** | **0.0343** |
| CD4+ IFNg | 0.195, n=14 | 0.299, n=27 | 0.4840 |
| CD4+ IL-2 | 0.173, n=14 | 0.149, n=27 | 0.5012 |
| CD4+ TNF | 0.4875, n=14 | 0.862, n=27 | 0.1824 |
| CD4+ IL-17 | 0.4655, n=14 | 0.339, n=27 | 0.2161 |
| CD4+ IL-10 | 0.189, n=9 | 0.303, n=17 | 0.4992 |
| CD4+ Ki67 | **0.0638, n=8** | **0.0929, n=15** | **0.0556** |
| CD8+ | 27.95, n=14 | 29.6, n=27 | 0.5362 |
| CD8+ IFNg | 0.2435, n=14 | 0.239, n=27 | 0.7915 |
| CD8+ IL-2 | 1.464, n=14 | 0.772, n=27 | 0.6200 |
| CD8+ TNF | 0.763, n=14 | 1, n=27 | 0.5635 |
| CD8+ IL-17 | 0.507, n=14 | 0.382, n=27 | 0.5962 |
| CD8+ IL-10 | 0.572, n=9 | 0.52, n=17 | >0.9999 |
| CD8+ Ki67 | **0.331, n=8** | **0.676, n=15** | **0.0337** |
