# Supplementary material for: Lymph nodes are sites of prolonged bacterial persistence during Mycobacterium tuberculosis infection in macaques
Source: PLoS Pathog. 2018 Nov 1;14(11):e1007337. doi: 10.1371/journal.ppat.1007337 (PMC6211753; doi:10.1371/journal.ppat.1007337)
Supplement: S5 Table — MCT = multiple comparison test. (DOCX) [file ppat.1007337.s013.docx]

| Cell Type/Cytokine | No effacement | | Effacement <50% | | >50% Effacement | | Kruskal Wallis with Dunns MCT |
| --- | --- | --- | --- | --- | --- | --- | --- |
|  | Mean | Std. Deviation | Mean | Std. Deviation | Mean | Std. Deviation | p value |
| CD3 (of Lymphocytes) | 78.1 | 11.67 | 75.5 | 8.271 | 70.8 | 8.585 | 0.0839 |
| CD3+IFNg | 1.38 | 3.029 | 0.76 | 0.8869 | 1.65 | 2.715 | 0.8182 |
| CD3+ IL-2 | 1.2 | 1.606 | 1.55 | 1.906 | 1.29 | 2.329 | 0.8494 |
| CD3+ IL-10 | 2.17 | 5.317 | 4.67 | 6.198 | 9.07 | 14.27 | 0.2515 |
| CD3+ IL-17 | 1.93 | 4.723 | 4.41 | 8.153 | 4.29 | 9.848 | 0.7276 |
| CD3+ Ki67 | 0.96 | 1.302 | 2.94 | 3.354 | 0.44 | 0.05873 | 0.7931 |
| CD3+ TNF | 1.3 | 1.484 | 2.2 | 3.774 | 1.18 | 0.9733 | 0.9433 |
| CD4 (of CD3+) | 57.6 | 16.01 | 60.7 | 12.25 | 59.9 | 7.813 | 0.7747 |
| CD4+ IFNg | 1.3 | 3.045 | 0.68 | 0.9098 | 1.64 | 2.76 | 0.9917 |
| CD4+ IL-2 | 0.84 | 1.745 | 1.21 | 1.999 | 1.12 | 2.495 | 0.6361 |
| CD4+ IL-10 | 2.25 | 5.92 | 4.91 | 8.376 | 9.74 | 16.88 | 0.456 |
| CD4+ IL-17 | 1.81 | 4.849 | 4.22 | 8.232 | 4.47 | 10.45 | 0.5937 |
| CD4+ Ki67 | 0.71 | 1.147 | 2.23 | 2.742 | 0.24 | 0.1443 | 0.5842 |
| CD4+ TNF | 1.38 | 1.537 | 1.98 | 3.742 | 1.19 | 1.051 | 0.5316 |
| CD8 (of CD3+) | 26.5 | 7.585 | 24.4 | 8.817 | 24.3 | 8.507 | 0.7167 |
| CD8+ IFNg | 1.5 | 3.56 | 0.87 | 0.9963 | 1.71 | 2.775 | 0.64 |
| CD8+ IL-2 | 1.88 | 1.952 | 2.09 | 2.211 | 1.71 | 2.481 | 0.8919 |
| CD8+ IL-10 | 1.21 | 2.662 | 3.06 | 4.768 | 5.05 | 7.011 | 0.841 |
| CD8+ IL-17 | 1.48 | 3.624 | 3.59 | 6.782 | 3.46 | 7.705 | 0.6135 |
| CD8+ Ki67 | 1.38 | 1.51 | 3.62 | 4.054 | 0.77 | 0.1641 | 0.7296 |
| CD8+ TNF | 1.16 | 1.509 | 2.23 | 3.729 | 1.02 | 0.8901 | 0.5074 |
